# CARP-1 functional mimetics are novel inhibitors of drug-resistant triple negative breast cancers

**DOI:** 10.18632/oncotarget.12333

**Published:** 2016-09-28

**Authors:** Vino T. Cheriyan, Magesh Muthu, Ketan Patel, Sreeja Sekhar, Walajapet Rajeswaran, Scott D. Larsen, Lisa Polin, Edi Levi, Mandip Singh, Arun K. Rishi

**Affiliations:** ^1^ John D. Dingell VA Medical Center, Wayne State University, Detroit, MI, 48201 USA; ^2^ Karmanos Cancer Institute, Wayne State University, Detroit, MI 48201 USA; ^3^ Department of Oncology, Wayne State University, Detroit, MI 48201 USA; ^4^ Department of Pathology, Wayne State University, Detroit, MI 48201 USA; ^5^ Vahlteich Medicinal Chemistry Core and College of Pharmacy, University of Michigan, Ann Arbor, MI 48109 USA; ^6^ College of Pharmacy and Pharmaceutical Sciences, Florida A&M University, Tallahassee, FL 32307, USA

**Keywords:** CFMs, resistant TNBCs, CARP-1, mammospheres, apoptosis

## Abstract

Doxorubicin and Cisplatin are the frontline therapeutics for treatment of the triple negative breast cancers (TNBCs). Emergence of drug-resistance often contributes to failure of drugs and poor prognosis, and thus necessitates development of new and improved modalities to treat TNBCs. We generated and characterized chemotherapy-resistant TNBC cells following their culture in chronic presence of Doxorubicin or Cisplatin, and tested whether their viabilities were inhibited by a novel class of CARP- 1 functional mimetic (CFM) compounds. Analogs of parent compound CFM-4 were obtained through structure-activity based medicinal chemistry studies. CFM-4.16, a novel analog of CFM-4, caused superior inhibition of viability of TNBC cells when used in combination with doxorubicin. Doxorubicin and cisplatin inhibited viabilities of parental cells with GI_50_ dose of 0.02–0.1 μM and 1.65 μM, respectively. The GI_50_ dose of doxorubicin for doxorubicin-resistant TNBC cells was ≥ 10.0 μM. For Cisplatin-resistant cells, the GI_50_ dose of Cisplatin was ≥ 6–15.0 μM for MDA-MB-468 sublines and ≥ 150.0 μM for MDA-MB-231 sublines. CFM-4.16 inhibited viability of chemotherapy-resistant TNBC cells, in part by inhibiting oncogenic cMet activation and expression, stimulating CARP-1 expression, caspase-8 cleavage and apoptosis. CFM-4.16 pretreatment enhanced anti-TNBC efficacies of inhibitors of cMET (Tevatinib) or cSrc (Dasatinib). CFM-4.16 suppressed growth of resistant TNBC cells in soft agar as well as in three-dimensional suspension cultures derived from enriched, stem-like cells. Finally, a nanolipid formulation of CFM-4.16 in combination with doxorubicin had superior efficacy in inhibiting TNBC xenograft growth. Our findings collectively demonstrate therapeutic potential of CFM-4.16 for parental and drug-resistant TNBCs.

## INTRODUCTION

The American Cancer Society estimates indicate approximately 246,000 new cases and 40,000 deaths in the United States resulting from breast cancers in females in 2016 [1]. Over the previous decade, the incidence rates and consequent mortality associated with the breast cancers decreased in part due to advances in diagnosis and therapeutic modalities. The development of therapeutics that target estrogen receptor (ER) function and estrogen biosynthesis, and the human epidermal growth factor receptor (EGFR) 2 (aka Her2) have benefited a vast majority of breast cancer patients. However, a significant percent of breast cancers lack ER, progesterone receptor (PR), and Her2, and are often grouped as triple-negative breast cancers (TNBCs). Chemotherapy including an anthracycline, cisplatin and/or taxane-based regimen remains the current best standard of care for TNBCs. A recent study indicating existence of molecular subtypes among TNBCs underscores further stratification of this subgroup of hard-to-treat cancers, and emphasizes the unmet need for identification of better molecular-based therapies [2]. Although a number of cell growth and survival pathways are being actively pursued for targeting TNBCs [3], better and effective strategies are urgently needed to overcome drug resistance and improve therapeutic outcomes.

Cell cycle and apoptosis regulator 1 (CCAR1/CARP-1) is a peri-nuclear phospho-protein, that regulates cell growth and apoptosis signaling in a variety of cancer cells [4–7]. In addition to transcriptional co-activation of the steroid family of nuclear receptors, CARP-1 regulates Doxorubicin/Adriamycin (ADR)-dependent DNA damage-induced apoptosis in a manner dependent as well as independent of co-activation of tumor suppressor p53 [4, 5]. Withdrawal of serum growth factors or blockage of EGFR results in elevated CARP-1 expression, cell cycle arrest, and apoptosis, while knockdown of CARP- 1 resulted in resistance to apoptosis by ADR or EGFR tyrosine kinase inhibitors [4–6].

In an attempt to elucidate molecular mechanisms of CARP-1 signaling, we performed yeast-two-hybrid assays and discovered that CARP-1 binds with cell cycle regulatory anaphase promoting complex/cyclosome (APC/C) E3 ligase subunit APC2 [8]. APC/C is a multi-subunit ubiquitin E3 ligase protein that functions to regulate ubiquitin-dependent proteasomal turnover of a large number of cellular proteins including the cell cycle regulatory cyclin B1, CDC20, Cdh1, and SCF E3 ligase. APC/C has been well-known to play a distinct role in cell cycle transitions [9, 10], and prior reports have shown that misregulation of APC/C and its substrates correlates with tumor progression [11]. We exploited the APC/C co-activation function of CARP-1 and identified a number of small molecule inhibitors (SMIs) of CARP-1 binding with APC2 [8]. These compounds, termed CARP-1 functional mimetics (CFMs), inhibit cell growth by inducing apoptosis in a variety of cancer cells [8, 12–14]. Here we provide evidence that CFMs are novel and potent inhibitors of drug-resistant TNBCs.

## RESULTS

### CFM-4.16, a novel CFM-4 analog, is a potent inhibitor of TNBC cells

**Figure 1:**
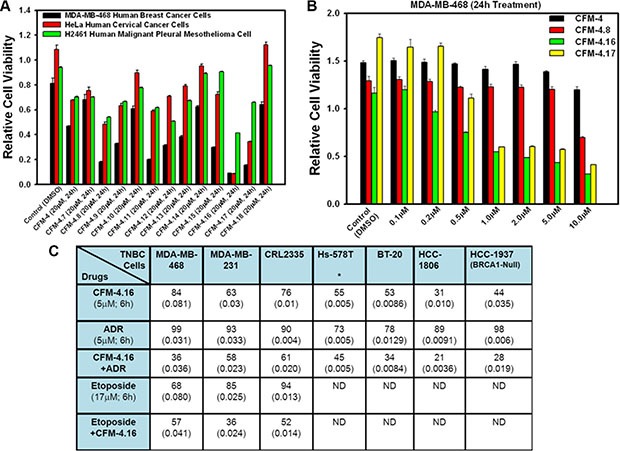
CFM-4.16 inhibits TNBC cell growth and enhances ADR efficacy (**A**, **B**), Noted cell lines were either treated with DMSO (Control), or with various CFMs for indicated dose and time. Cell viability was determined by MTT assay. The data in the histograms represent means of three independent experiments; bars, S.E. (**C**) Indicated TNBC cells were treated either with DMSO, CFM-4.16, ADR, etoposide, ADR plus CFM-4.16, or etoposide plus CFM-4.16, and percent cell viabilities were determined relative to DMSO-treated controls. *, In the case of Hs-578T cell, the CFM-4.16 dose was 100 nM; (), SEM.

Our previous studies have indicated cancer cell growth inhibitory properties of CFMs in particular CFM-4 and CFM-5 [8]. These compounds although were soluble in DMSO, the intravenous administration of DMSO+cremophor preparations of CFM-4 failed to inhibit growth of xenografted TNBC tumors in SCID mice [12]. A nanolipid formulation of CFM-4 (CFM-4 NLF) however resulted not only in increased systemic bioavailability of this compound, the oral administration of this NLF inhibited growth of orthotopically transplanted xenografts of human TNBC as well as non-small cell lung (NSCLC) cancer cells [12]. On the basis of these findings we speculated that solubility and/or potency of the CFM-4 scaffold could be improved for its utility as a novel anti-TNBC molecule. To address this possibility, we first conducted medicinal chemistry based structure activity relationship (SAR) studies and synthesized 12 additional analogs of CFM-4 (Table 1). Each of the analog was suspended in DMSO, and their potency was evaluated in cell culture studies utilizing HeLa cervical cancer, MDA-MB-468 TNBC, and H2461 malignant pleural mesothelioma (MPM) cells by MTT based assays. As shown in Figure 1A, CFM-4.8, −4.11, −4.16, and −4.17 elicited greater inhibition of viability of TNBC cells when compared with CFM-4 at the tested dose of 20 μM for 24 h period for each compound. Interestingly, CFM-4.16 and −4.17 compounds caused greater inhibition of viability of HeLa cells while only CFM-4.16 caused a greater loss of viability of MPM cells when compared with CFM-4 at the tested doses of 20 μM for 24 h period for each compound. Moreover, a 10 μM dose of CFM-4 inhibited MDA-MB-231 and MDA-MB-468 viabilities by ~30% and 45%, respectively. A comparable, 10 μM dose of CFM-4.16 however resulted in ≥ 80–100% loss of cell viability of either of the TNBC cells (not shown), suggesting that CFM-4.16 was likely more potent analog for TNBC cells. Further dose response analysis revealed that either of the CFM-4.16 or −4.17 inhibited TNBC cell viabilities by 50% or higher at the doses of ≥ 1 μM (Figure 1B). Additional dose response analyses revealed that although the CFM-4.16 dose for inhibition of the TNBC cells growth by 50% (GI_50_) was ~2 μM, since CFM-4.16 also stimulated apoptosis (see below), its dose for inducing a 50% cytotoxic effects (LC_50_) was ~7–8 μM (not shown).

**Table 1:** List and chemical modifications of CFM-4 analogs 4.7–4.18

CFM	R^1^	R^2^	R^3^	R^4^
4 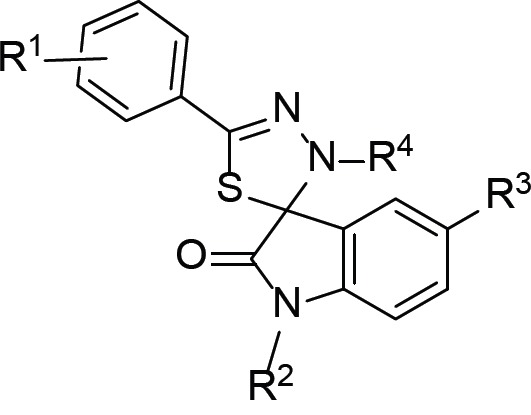	H	2-Cl-Ph-CH_2_	H	H
4.7	H	4-Cl-Ph-CH_2_	H	H
4.8	H	2-napthyl-CH_2_	H	H
4.9	H	3-Cl-Ph-CH_2_	H	H
4.10	H	2-pyr-CH_2_	H	H
4.11	H	2-Cl-Ph-CH_2_	MeO	H
4.12	H	2-MeO-Ph-CH_2_	H	H
4.13	H	8-quinolinyl-CH_2_	H	H
4.14	2-CH_3_	2-Cl-Ph-CH_2_	H	H
4.15	H	2-Cl-Ph-CH_2_	Cl	H
4.16	3-Cl	2-Cl-Ph-CH_2_	H	H
4.17	3-OCH_3_	2-Cl-Ph-CH_2_	H	H
4.18	H	2-Cl-Ph-CH_2_	H	CH_3_

Given that the frontline anti-TNBC therapeutic ADR has a molecular mass of 543.5, and the molecular mass of CFM-4.16 is 440.3, we next tested whether and to what extent an equimolar dose of each compound will inhibit growth of the TNBC cells. For this purpose, a number of TNBC cells were treated with 5 μM dose of each agent separately or in combination for a short duration of 6 h. This experiment interestingly revealed that although each agent caused inhibition of viability of all the TNBC cells, CFM-4.16, but not CFM-4 or any of its other analogs, elicited a greater loss of cell viability of each cell line when compared with the respective, ADR-treated cell line (Figure 1C). CFM-4.16 also enhanced ADR-mediated inhibition of viability of TNBC cells (Figure 1C). CFM-4.16 however, failed to enhance ADR- mediated growth inhibition of a variety of other cancer cell lines tested including the MCF-7, SK-BR-3 and MDA-MB-453 breast cancer cells (not shown). The precise mechanism(s) of increased inhibition of TNBC cell growth by a combination of CFM-4.16 and ADR are yet to be clarified. Our prior studies have shown that ADR or CFM compounds function in part by stimulating CARP-1 levels, and CARP-1 expression was found necessary for apoptosis signaling by ADR as well as by CFM-4 [4, 5, and 8]. It is likely then that TNBC cell growth inhibitory signaling by CFM-4.16 involves mechanisms that are overlapping as well as distinct from those utilized by ADR, and the fact that the emergence of drug (ADR or Cisplatin) resistant TNBCs remain a significant and unresolved clinical problem, provided us with a rationale to explore whether CFM-4.16 would be suitable for inhibiting growth of drug-resistant TNBC cells.

To test whether CFM-4.16 compound will be an effective inhibitor of chemotherapy (ADR or Cisplatin)-resistant TNBC cells, we first generated a number of stable, TNBC sublines/clonal derivatives that were cultured in prolonged, chronic presence of ADR or Cisplatin essentially as detailed in methods. The parental and resistant sublines were then separately subjected to MTT-based assays for determination of their respective GI_50_ dose for ADR or Cisplatin (Table 2). Of note is the fact that although the GI_50_ dose of Cisplatin and ADR for the cisplatin-resistant MDA-MB-231 cells and the ADR-resistant MDA-MB-468 cells was ≥ 90-fold and ≥ 500-fold, respectively, higher than their wild-type, parental counterparts, the GI_50_dose of Cisplatin for the cisplatin-resistant MDA-MB-468 cells however was only 4–9-fold higher than their wild-type, parental cells (Table 2). We next utilized these drug-resistant cells to determine whether CFM-4.16 inhibits their growth and investigated the molecular mechanisms involved as detailed below.

**Table 2:** GI_50_ values of parental and drug-resistant TNBC cells

Cell Line	Cisplatin (72 h)		Cell Line	Adriamycin (72 h)	
		GI_50_(μM)			GI_50_(μM)
MDA-MB-468	Wild type	~1.65	MDA-MB-468	Wild type	~0.02
	Cis-R Clone 1–2	~6.6		ADR-R Clones 1–6	~10.0
	Cis-R Clone 3	~12.0	4T1	Wild type	~0.1
	Cis-R Clones 4–6	~15.0		ADR-R Clones 1–6	> 10.0
MDA-MB-231	Wild type	~1.65	MDA-MB-231	Wild type	< 0.1
	Cis-R Clones 1–6	≥ 150.0		ADR-R Clones 1–6	> 10.0

In the case of Adriamycin-resistant cells, the respective parental and resistant (ADR-R) sublines were treated with 0.1, 0.2, 1.0, 2.0, 4.0, and 10.0 μM dose of ADR. In the case Cisplatin-resistant cells, the respective parental and resistant (Cis-R) sublines were treated with 1.65, 3.30, 6.60, 16.50, 33.00, and 66.00 μM dose of Cisplatin. Percent cell viabilities were determined relative to respective DMSO-treated controls. The data in the GI_50_ columns represent means of three independent experiments

**Figure 2:**
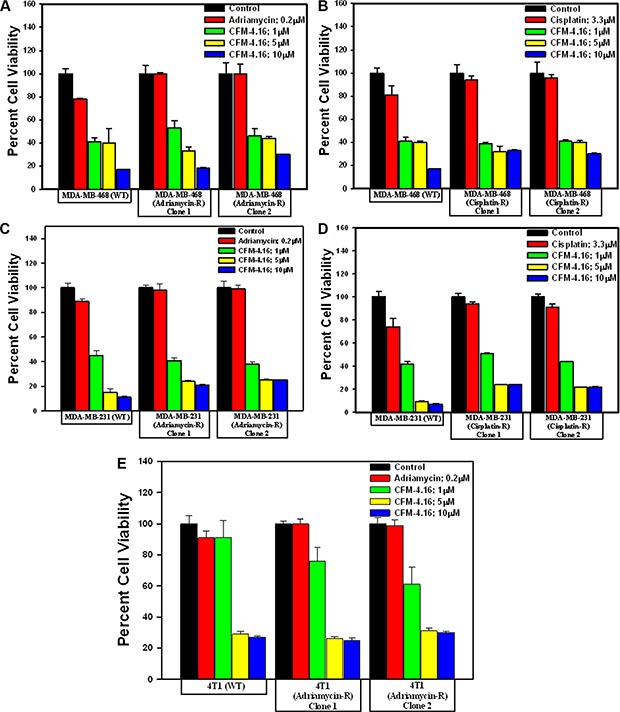
CFM-4.16 inhibits drug-resistant TNBC cell growth in dose-dependent manner (**A–E**) Indicated parental and their respective drug resistant TNBC cells were either untreated (Control) or treated with noted doses of Adriamycin or CFM-4.16 for 12 h. Cell viability was determined by MTT assay. The histogram columns represent means of three independent experiments; bars, S.E.

First, the parental as well as the respective drug-resistant sublines were exposed to various doses of CFM- 4.16 followed by determination of their viability by MTT-based assays as in methods. Since the GI_50_ doses for ADR and Cispaltin for 72 h treatment period were < 0.1 and ~1.65 μM, respectively, for both the parental TNBC cell lines (Table 2), a dose of 0.2 μM ADR or 3.3 μM Cisplatin was chosen for a shorter, 12 h treatment periods to avoid extensive loss of cell viability and/or cell death. As shown in Figure 2, CFM-4.16 inhibited growth of both the wild-type TNBC cells as expected. CFM-4.16 also effectively inhibited growth of the ADR or Cisplatin-resistant TNBC sublines in a dose-dependent manner, suggesting that CFM class of scaffolds could be novel precursors of molecules for treatment of TNBCs including their drug-resistant variants.

### CFM-4.16 promotes apoptosis in TNBC cells by activating p38 MAP kinase, c-Jun N-terminal kinase (JNK) and stimulating expression of CCAR-1/CARP-1

**Figure 3:**
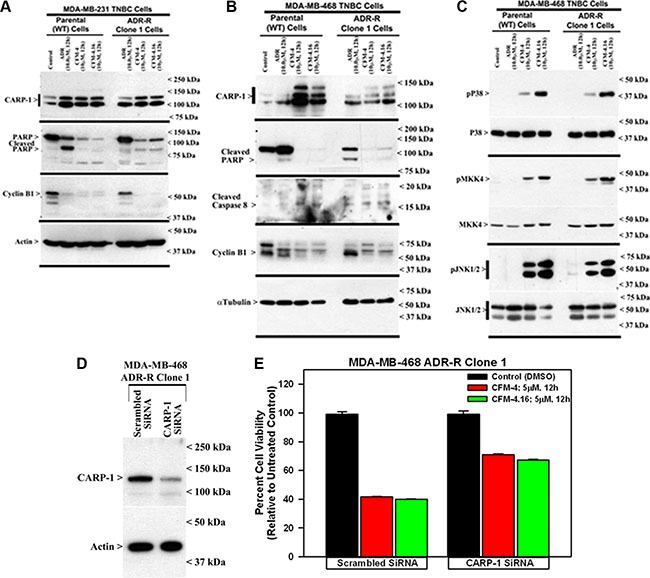
CFM-4.16 stimulates apoptosis in parental and ADR-resistant TNBC cells in part by upregulating pro-apoptotic CARP-1 and activating SAPKs Indicated TNBC cells were either untreated (Control), treated with ADR, CFM-4, or CFM-4.16 for noted dose and time. Cell lysates were analyzed by Western blotting (WB) as in Methods for levels of CARP-1, cyclin B1, cleaved PARP and caspase-8 (**A**, **B**) and activation (phosphorylation) of pro-apoptotic p38, MKK4, and JNK1/2 SAPKs (**C**). Knockdown of CARP-1 blocks CFM-4.16 effects. Cells were transfected with 100 nM each of the scrambled or CARP-1 siRNAs (**D**, **E**) for 72 h and were then either untreated (Control/DMSO), treated with CFM-4 or CFM-4.16 for noted time and dose. Cell lysates were subjected to WB as in panel B above (D) or to MTT assay for determination of cell viabilities as in Figure 2 (E). The histogram columns represent means of two independent experiments; bars, S.E.

CARP-1 has previously been shown to function as a regulator of breast cancer cell growth by ADR [4–6]. Knock-down of CARP-1 resulted in elevated levels of topoisomerase IIα [4], and abrogated HBC cell growth inhibition by ADR, Etoposide or CFM-4 [4, 5, and 8]. Moreover, CFM-4 and its analog CFM-4.6 inhibited growth of TNBC and NSCLC cells in part by inducing apoptosis and stimulating activation of pro-apoptotic, stress-activated protein kinases (SAPKs) p38α/β and JNK1/2, caspase-8, and cleavage of PARP [12]. Consistent with these findings, our western blot analyses in Figure 3A–3C and Supplementary Figure 1 show that CFM-4.16 activated pro-apoptotic SAPKs p38α/β and JNK1/2, caspase-8, while causing cleavage of PARP and decline in mitotic cyclin B1 levels in both the parental and drug-resistant TNBC cells. Since prior studies have demonstrated a requirement for CARP-1 expression in transduction of growth inhibitory signaling by ADR or CFM-4 [4, 5, 8], we next determined whether CARP- 1 was also required for CFM-4.16-dependent growth inhibition of drug-resistant TNBC cells. For this purpose, we conducted siRNA-mediated knock-down of CARP-1 in ADR-resistant MDA-MB-468 subline 1 cells essentially following methods described by us before [8]. SiRNA-mediated depletion of CARP-1 levels in ADR-resistant MDA-MB-468 subline 1 cells (Figure 3D) interfered with growth inhibition of these cells by CFM-4 or CFM-4.16 (Figure 3E). These data suggest that CARP-1 is a necessary transducer of inhibitory signaling by CFM-4 and its analog CFM-4.16 in the parental as well as the drug-resistant TNBC cells. It is of note here that CFM-4.16 treatment induced a rather robust activation of both the pro-apoptotic SAPKs in comparison with CFM-4 in the parental as well as resistant TNBC cells (Figure 3C). Whether and to the extent such robust activation of pro- apoptotic SAPKs by CFM-4.16 contributes to its superior TNBC growth inhibitory effects remain to be clarified.

### CFMs inhibit activation and/or expression of cell growth and survival promoting oncogenic tyrosine kinases

**Figure 4:**
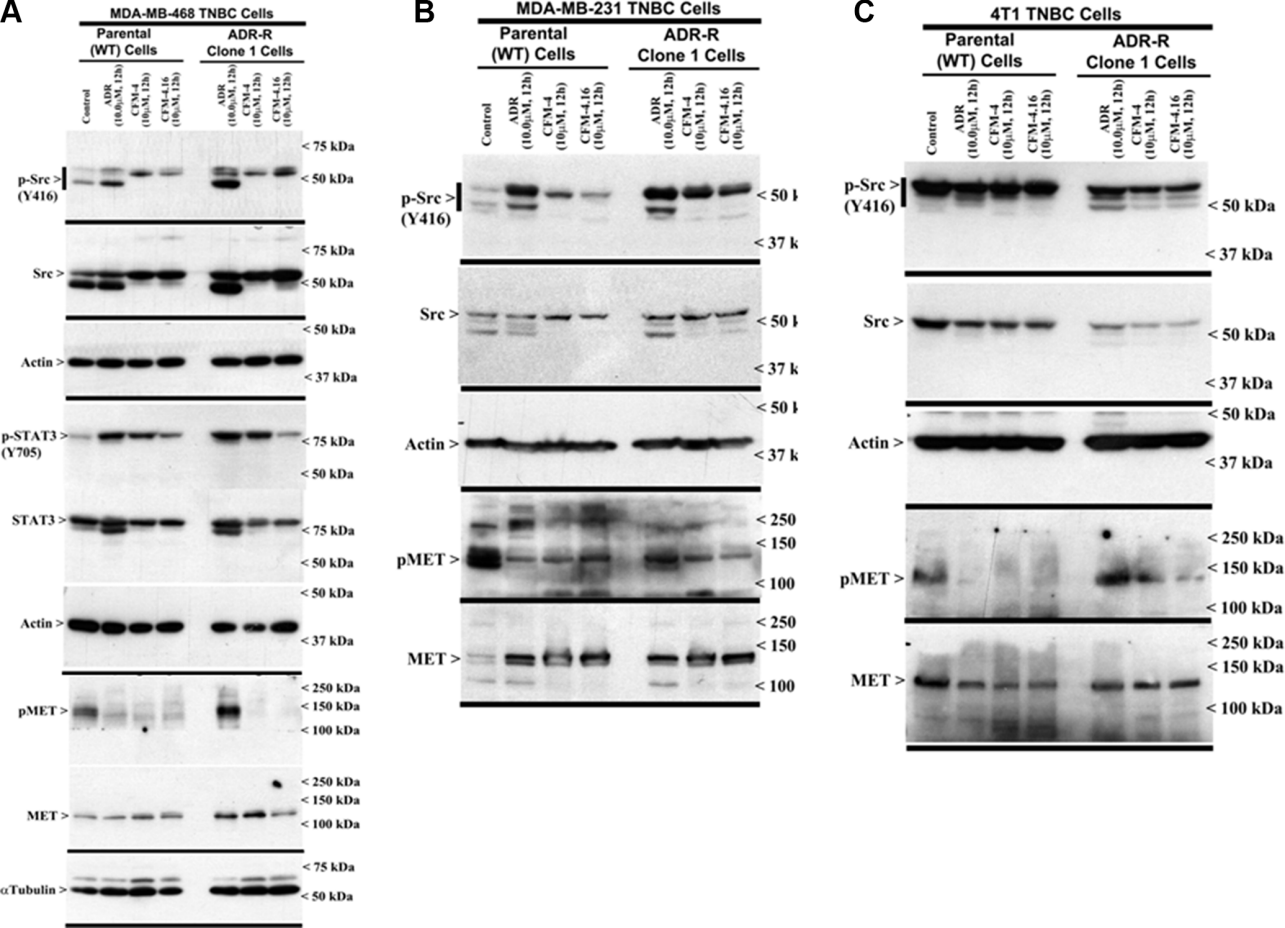
CFM-4.16 inhibits oncogenic tyrosine kinases in ADR-resistant TNBC cells (**A–C**) Indicated TNBC cells were either untreated (Control), treated with ADR, CFM-4, or CFM-4.16 for noted dose and time. Cell lysates were analyzed for expression and activation (phosphorylation) of Src, MET, and STAT3 kinases, and levels of actin and α-tubulin proteins by Western blotting as described in Methods. Identity of respective protein and molecular weight markers is denoted by arrowheads on the left and right side, respectively, of each WB.

Aberrant expression and/or activation of oncogenic signaling by kinases such as the Src and Abl tyrosine kinases, and receptor tyrosine kinases (RTKs) MET, Vascular endothelial growth factor receptor (VEGFR), Fibroblast growth factor receptor (FGFR), Insulin-like growth factor receptor 1 (IGF-1R), as well as members of the epidermal growth factor receptor (EGFR) family, have been well documented to play pivotal roles in cancer development, progression, metastasis, and often function as significant drivers of development of therapy resistance in many cancers including TNBCs [15]. We conducted western-blot analyses to further elucidate molecular mechanisms of TNBC growth inhibition by CFM-4 and its analog CFM-4.16, and to determine whether CFM class of compounds targeted activation and/or expression of oncogenic tyrosine kinases. The parental and drug-resistant TNBC cells were treated with ADR, CFM-4 or CFM-4.16, and in the first instance, the cell lysates were analyzed for activation and/or expression of Src and MET tyrosine kinases. As shown in Figure 4 and Supplementary Figures 2 and 3, expression and/or activity of MET RTK was moderately elevated in ADR as well as Cisplatin-resistant TNBC cells. Activity and/or expression of Src were also moderately elevated in ADR-resistant human TNBC cells but not in ADR-resistant murine or cisplatin-resistant human TNBC cells. These data are consistent with well-documented roles of these oncogenic kinases as drivers of drug resistance in many cancers including TNBCs. CFM-4 and CFM-4.16 treatments however caused reduced activation and/or expression of both MET and Src tyrosine kinases in parental as well as drug-resistant human and murine TNBC cells (Figure 4 and Supplementary Figure 2), suggesting that CFM class of molecules function in part by targeting oncogenic kinases and their signaling to promote apoptosis and suppress cell growth. Interestingly, activation of STAT3, a well-known down-stream transducer of signaling by activated EGFR and Src tyrosine kinases in TNBC cells [16], was robustly attenuated in CFM-4.16 but not CFM-4-treated ADR as well as Cisplatin-resistant human TNBC cells (Figure 4A and Supplementary Figure 2C). These findings together with our data demonstrating a robust activation of pro-apoptotic SAPKs by CFM-4.16 when compared with CFM-4 would suggest for potential of this compound as a superior inhibitor of drug-resistant TNBCs.

**Figure 5:**
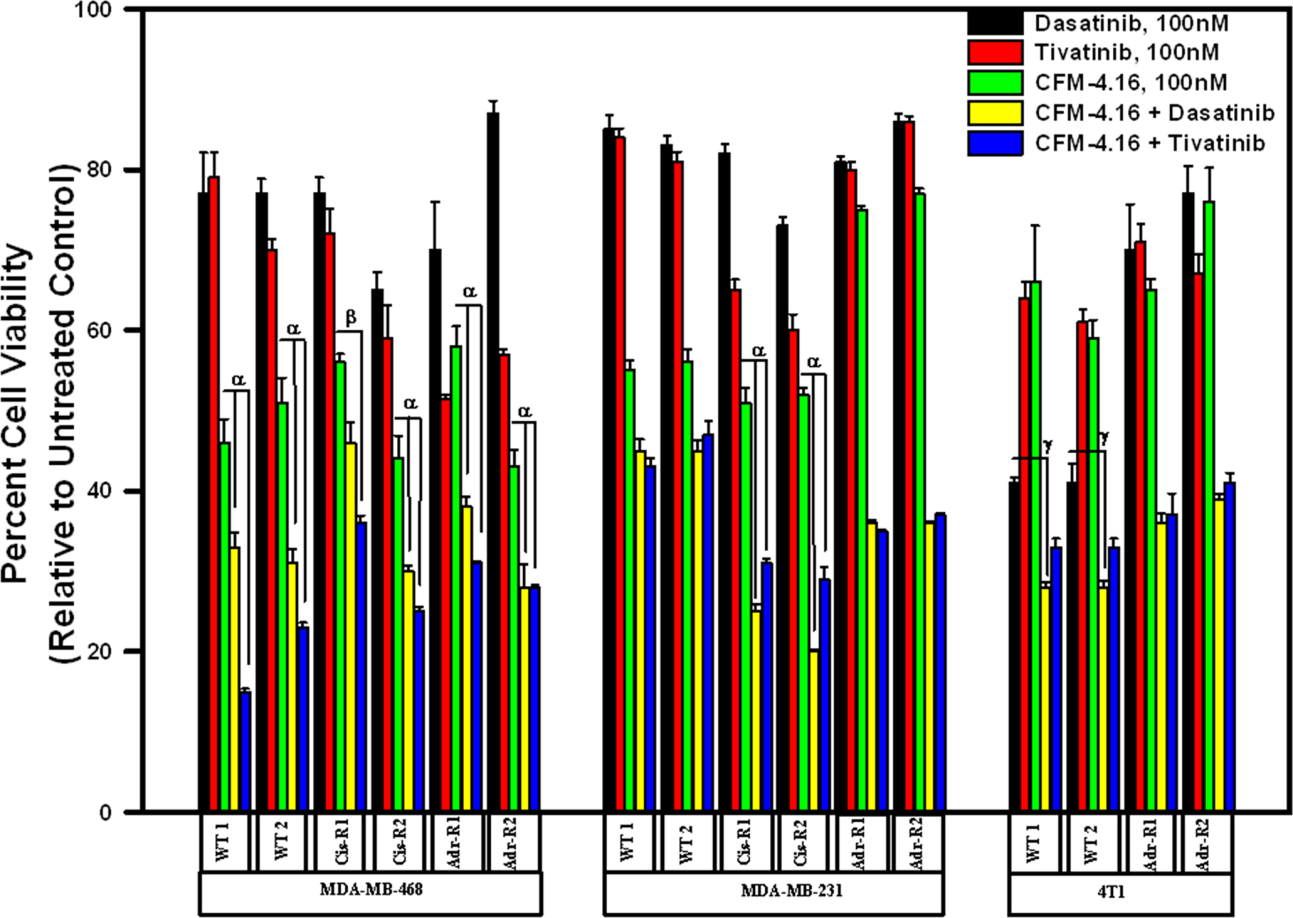
CFM-4.16 enhances efficacy of compounds that target MET or Src kinases in drug-resistant TNBCs Indicated parental and their respective drug-resistant sublines were untreated, treated with noted dose of MET inhibitor Tevatinib, Src inhibitor Dasatinib, CFM-4.16, a combination of CFM-4.16 and Dasatinib, or a combination of CFM-4.16 and Tevatinib. Please note that the cells were exposed to CFM-4.16 for 24 h while cells were treated with Dasatinib or Tevatinib (as single agent or in combination with CFM-4.16) for 12 h. Cell viability was determined by MTT assay as in Figure 1. The histogram columns represent means of three independent experiments; bars, S.E. α and β, *p* = < 0.03 relative to respective cells treated with CFM-4.16 only. γ, *p* = < 0.01 relative to respective cells treated with Dasatinib only.

Since MET and Src tyrosine kinases are moderately activated and/or overexpressed in drug resistant TNBC cells and CFM-4.16 treatments attenuated activation of MET in parental and drug-resistant TNBC cells, while impacting Src activities only in Cisplatin-resistant TNBC cells (Figure 4 and Supplementary Figure 2), we next determined whether pre-treatment with CFM-4.16 could sensitize/enhance growth suppression of TNBC cells by pharmacologic inhibitors of MET and/or Src kinases. For this purpose, we utilized Dasatinib that is a multi-targeted orally administered inhibitor of RTKs and Src family of tyrosine kinases [17] that is FDA approved treatment for chronic myelogenous leukemia (CML). In addition, we utilized Tivatinib, an investigational orally administered, highly selective inhibitor of the MET RTK [18]. The parental and the drug-resistant TNBC cells were either treated with CFM-4.16, Dasatinib, or Tivatinib as single agents or the cells were first treated with CFM-4.16 for 12 h, followed by addition of Dasatinib or Tivatinib for another 12 h. As shown in Figure 5, treatments with Dasatinib or Tivatinib alone generally elicited a moderate, 20–40% loss of viability of parental as well as drug-resistant human TNBC cells. Although parental and drug-resistant human TNBC cells, with the exception of ADR-resistant MDA-MB-231 cells, elicited a higher 45–55% loss of viability when exposed to CFM-4.16 alone, CFM-4.16 in combination with Tivatinib provoked a much greater loss of viabilities of these cells when compared with either agent alone. Dasatinib in combination with CFM-4.16 however was more effective in inhibiting viabilities of drug-resistant MDA-MB-231 cells when compared with loss of viabilities elicited following treatments with either agent alone. Surprisingly, while Tivatinib or CFM-4.16 caused a reduction of ~30–40% viability of murine wild-type 4T1 TNBC cells, and Dasatinib caused ~60% loss of viability of these cells, either of these compounds caused a moderate, 20–30% loss of viability of the ADR-resistant 4T1 cells. A combination of Tivatinib and CFM-4.16 caused much greater loss of viability of wild-type 4T1 cells, while a combination of CFM-4.16 with Dasatinib or Tivatinib elicited a much greater loss of viability of ADR-resistant 4T1 cells. Together these data support our hypothesis that low-dose combination of CFM-4.16 with MET targeting could be an effective approach for TNBCs including their drug-resistant counterparts.

### CFMs suppress migration and three-dimensional growth of the parental and drug-resistant TNBCs

We next investigated whether CFM-4.16 inhibited TNBC cell migration and growth as colonies in soft agar and 3-dimensional cultures *in vitro*. In addition, an *in vitro* tubule formation assay was conducted to determine anti-angiogenic properties of CFM-4.16. As shown in Supplementary Figure 4A, although CFM-4 or CFM-4.16 caused disruption of tubule formation by HUVECs when compared with untreated control, a rather robust disruption in tubule integrity was noted for CFM-4.16-treated HUVECs. Moreover, treatments with CFM-4 or CFM-4.16 prevented the parental as well as drug (ADR- or cisplatin-) resistant TNBC sublines and the parental and Herceptin-resistant, Her-2-positive SKBR-3 cells from growing in the areas of wound caused by a scratch (Supplementary Figures 4B, 4C, 5A, 5B, and 6A, 6B). CFM-4 or CFM-4.16 also caused significant reduction in size and number of colonies formed by the parental as well as drug (ADR- or cisplatin-) resistant TNBC or Herceptin-resistant, Her-2-positive SKBR-3 cells in soft agar (Supplementary Figures 4D, 5C, 5D, and 6C).

**Figure 6:**
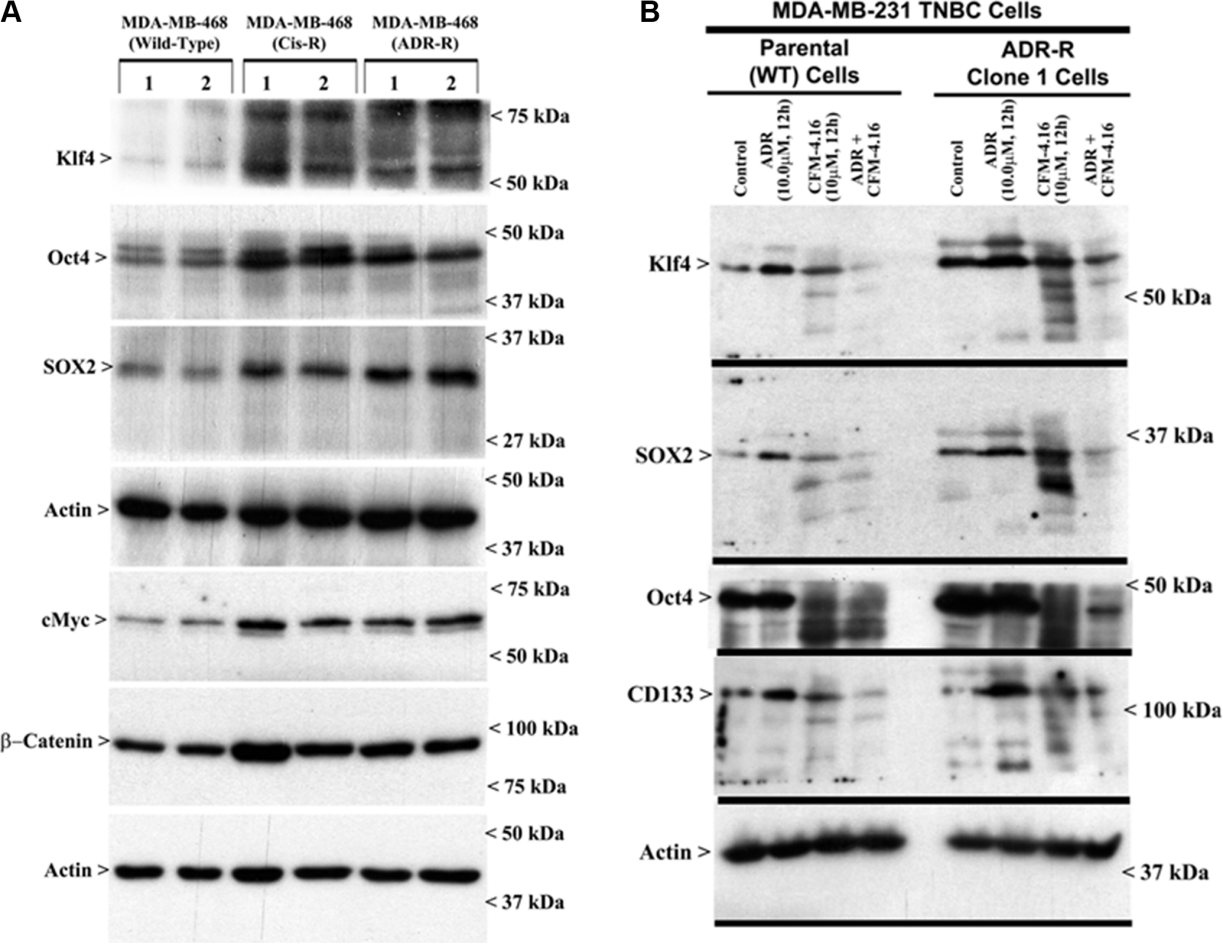
Drug-resistant TNBC cells have elevated expression of cancer stem cell genes, while CFM-4.16 in combination with ADR inhibits cancer stem cell gene expression Parental or drug-resistant TNBC cells were either untreated (**A**, **B**), treated with noted time and dose of indicated agent (B), and cell lysates were analyzed by Western blotting for levels of Klf4, Oct4, SOX2, CD133, cMyc, β-catenin and actin proteins as indicated in Methods. Identity of respective protein and molecular weight markers is denoted by arrowheads on the left and right side, respectively, of each WB.

A wealth of recent studies have indicated that a unique, small subpopulation of tumor cells have stem cell properties, which are often referred to as cancer stem-like cells (CSCs), that are capable of propagating the tumor as well as contribute towards development of resistance against conventional therapeutic drugs [19, 20]. The CSCs are often characterized by aberrant presence and/or expression of a number of distinct membrane and intracellular markers in various tumors [21]. Since CSC-associated markers for breast cancers include CD44, ALDH, EpCAM, CD133, ABCG2, Oct4, Sox2, Nanog, and Klf4, we first determined whether expression of any of these CSC-associated markers was altered in our drug-resistant TNBC cells, and to the extent their expression was impacted by CFM-4.16. Western-blot analysis revealed that expression of Klf4, Oct4, Sox2, c-Myc, and β-catenin was upregulated in ADR- or cisplatin-resistant MDA-MB-468 TNBC cells when compared with their parental counterparts (Figure 6A). Similarly, although expression of Klf4, Oct4, and Sox2 was also elevated in ADR-resistant MDA-MB-231 TNBC cells, treatment with CFM-4.16 caused a robust decline in levels of Oct4 in both the parental and ADR-resistant MDA-MB-231 TNBC cells (Figure 6B). A combination of ADR and CFM-4.16 however was highly effective in causing diminished levels of Klf4, Sox2, Oct4, and CD133 in both the parental and ADR-resistant MDA-MB-231 TNBC cells (Figure 6B). The data in Figure 6 collectively suggest that drug-resistant TNBC cells likely have a subpopulation of stem-like cells with elevated expression of CSC-associated markers that contribute to their growth and survival, and superior TNBC growth inhibition by ADR plus CFM-4.16 noted in Figure 1C could be due, in part, to their ability to target expression of different CSC-associated markers in the parental as well as drug-resistant TNBC cells.

**Figure 7:**
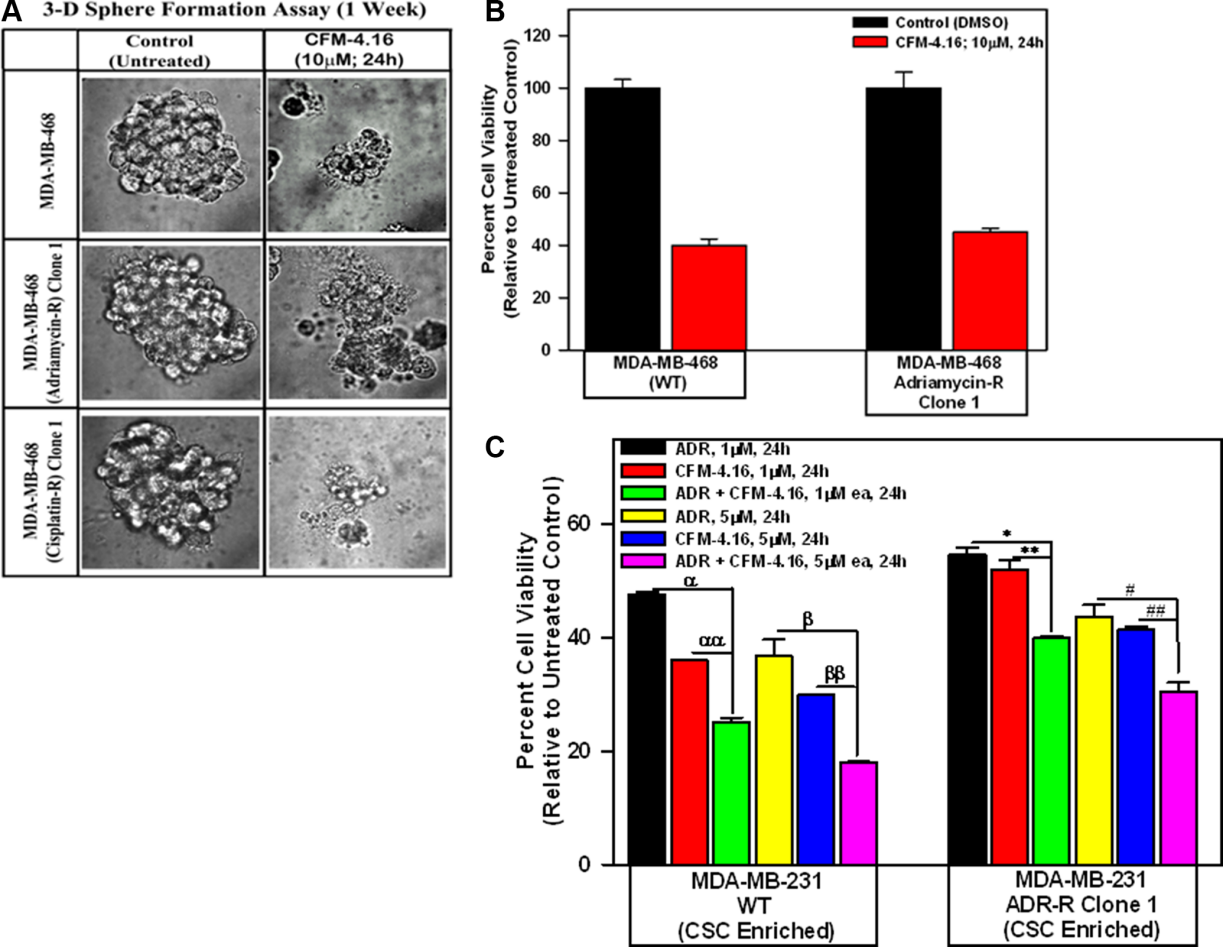
CFM-4.16 inhibits growth of mammospheres derived from parental and drug-resistant TNBC cells, and enhances efficacy of ADR in parental and ADR-resistant tumor-derived, CSC-enriched cells Parental and drug-resistant MDA-MB-468 TNBC cells were grown as mammospheres as detailed in Methods. The mammosphere cultures were either untreated (Control) or treated with CFM-4.16 for noted dose and time. The untreated and treated mammospheres were then photographed (**A**) or the cells were subjected to MTT-based viability assay as in Figure 1 (**B**). Representative photomicrographs of untreated and CFM-4.16 treated mammospheres are shown in panel A. C, The tumor-derived cells from xenografts of parental and ADR-resistant TNBC cells were enriched for CSCs as described in Methods. The CSC-enriched cells were then treated with ADR, CFM-4.16, or a combination of both for noted dose and time. The cell viabilities were determined by MTT-based assay as in Figure 1, and plotted relative to the MTT values for the respective untreated controls. The histogram columns in panels B and C represent means of three and four independent experiments, respectively; bars, S.E. α, αα, β, ββ, ^*, **, #, ##^*p* = < 0.03 relative to respective cells treated with ADR + CFM-4.16.

We next clarified whether and to the extent CFM-4.16 was able to interfere with growth of mammospheres derived from parental and drug-resistant TNBC-cells. In the first instance, mammospheres were grown from the 2-D cultures of parental and drug-resistant MDA-MB-468 TNBC cells as detailed in methods. The growing mammosphere cultures were then exposed to CFM-4.16, and the viabilities of untreated and treated cultures were determined by an MTT-based assay. Presence of CFM-4.16 caused disintegration of mammospheres of both the parental and drug-resistant MDA-MB-468 TNBC cells (Figure 7A). MTT assays revealed a robust decline in viability of CFM-4.16-treated mammospheres of parental as well as ADR-resistant cells when compared with their respective DMSO-treated controls (Figure 7B). Next, we utilized CSC enriched populations derived from xenografts of parental and ADR-resistant MDA-MB-231 TNBC cells to determine their inhibition by ADR, CFM-4.16, and a combination of both the agents. As shown in Figure 7C, either ADR or CFM-4.16 caused significant loss of viabilities of parental as well as ADR-resistant CSC enriched TNBC cells in a dose-dependent manner when compared with their untreated counterparts. Of note here is that CSC enriched populations derived from either the parental or ADR-resistant TNBC cells had significantly higher decline in their viabilities following exposure to a combination of CFM-4.16 and ADR when compared with the cells that were treated with either agent alone. Moreover, the parental CSC enriched cells treated with ADR, CFM-4.16, or a combination of both the agents generally had a greater decline in their viabilities when compared with the viabilities of similarly treated ADR-resistant CSC enriched cells (Figure 7C). The increased survival of CFM-4.16 or ADR-treated, ADR-resistant CSC enriched cells when compared with similarly treated parental CSC enriched cells noted in Figure 7C could be due in part to elevated levels of several CSC-associated markers in the ADR-resistant cells (see Figure 6) that are often well known to contribute to emergence, survival, and maintenance of drug resistance in TNBC and other cancers.

### Oral administration of CFM-4.16 NLF in combination with intravenous ADR causes superior inhibition of xenografted TNBC tumors

**Figure 8:**
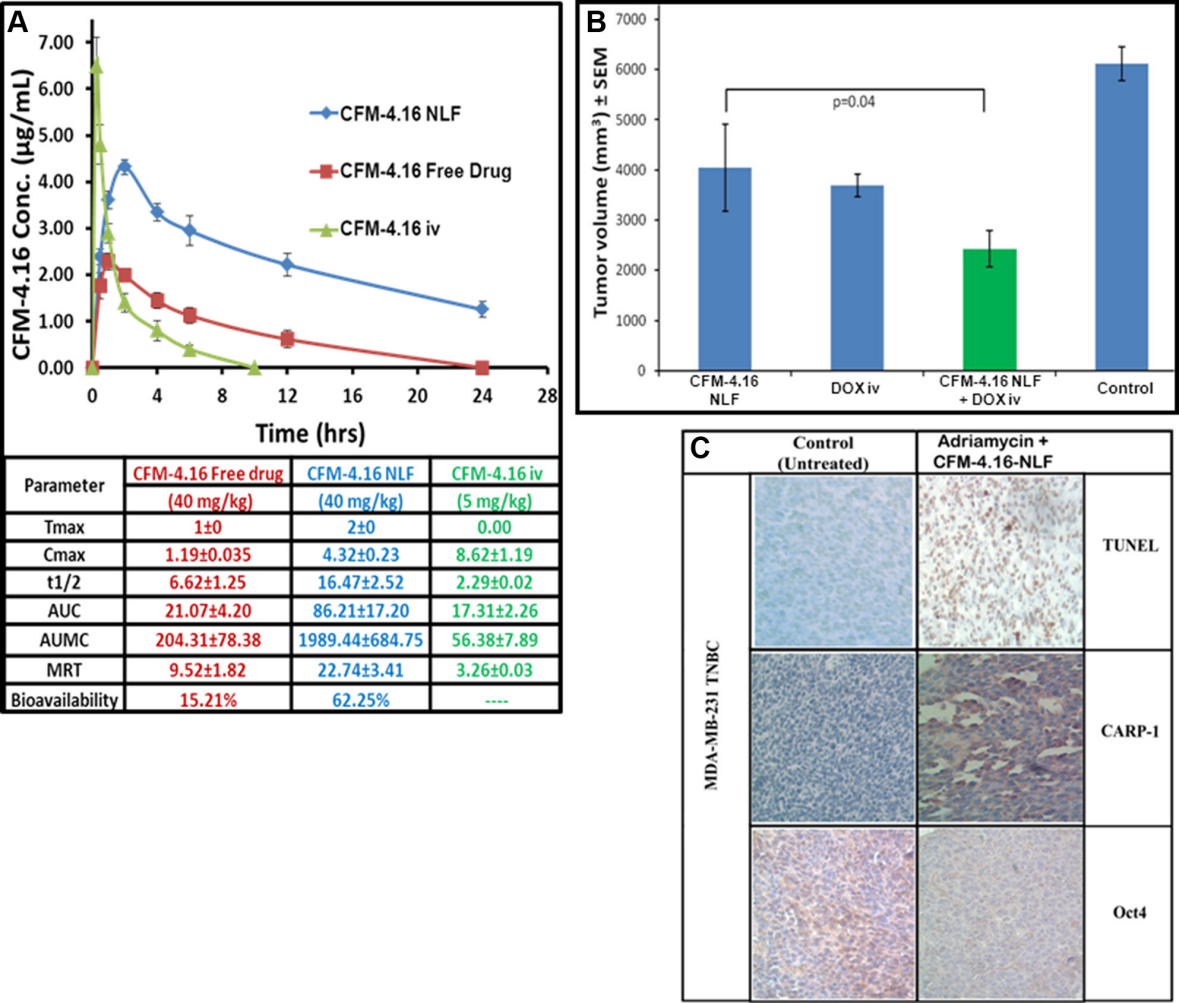
Formulation of surface modified CFM-4.16 NLF and evaluation of its pharmacokinetic parameters (A) and inhibition of TNBC cell-derived xenografts (B, C) (**A**) HPLC analysis of rat serum levels of CFM-4.16 at the noted time intervals following oral administration of indicated dose of CFM-4.16 NLF, CFM-4.16 Free drug, or intravenous (iv) administration of CFM-4.16 (noted as CFM-4.16 iv). Table in the lower part of panel A shows indicated pharmacokinetic parameters for CFM-4.16 when administered orally as CFM-4.16 free drug, CFM-4.16 NLF, or CFM-4.16 iv. Data analysis and calculations were performed essentially as detailed in Methods. (**B**) Histogram showing breast tumor volume of the placebo-treated (indicated as Control), CFM-4.16 NLF, Doxorubicin (indicated as DOX iv), or CFM-4.16 NLF + doxorubicin (indicated as CFM-4.16 NLF + DOX iv) treated, TNBC (MDA-MB-231) xenograft-bearing animals. The xenograft establishment, treatment and analysis procedures were carried out essentially as detailed in Methods. The columns represent average values from a total of six animals in respective group, bars, SE, significant where *p = 0.04 vs CFM NLF. (**C**) CFM-4.16 NLF plus Adriamycin treatments inhibit Oct4 expression, and induce CARP-1 expression and apoptosis in TNBC tumor xenografts. TNBC tumor xenografts generation and animal treatments were as in Methods. A representative tumor tissue from the placebo-treated (noted as Control) or CFM-4.16 NLF plus Adriamycin-treated animal was fixed in formalin, paraffin embedded, processed, and subjected to immuno-staining as detailed in Methods. Photomicrographs (400 × magnification) are shown for apoptosis (by TUNEL assay), and levels CARP-1 and Oct4 proteins as noted in methods. Elevated apoptosis is indicated by increased brown staining or dark-brown spots in CFM-4.16 NLF plus Adriamycin panels stained with anti-CARP-1 antibodies or TUNEL, respectively.

To investigate therapeutic potential of CFM-4.16, subcutaneous tumor xenografts derived from MDA-MB-231 TNBC cells were generated in NCR SCID mice and efficacy and potency of CFM-4.16 was first tested by intra-venous (Tail vein injection) administration as detailed in methods. This experiment failed to yield a therapeutic T/C values for this agent (Not shown). Since the xenograft studies involved intravenous administration of CFM-4.16 that was dissolved in DMSO plus cremophor, and a similar preparation of parent compound CFM-4 was previously also found to lack therapeutic T/C values in multiple xenograft studies [12], we suspected that systemic metabolism of these compounds likely contributed to their lower levels in serum that could have resulted in their lack of xenograft inhibitory effects. However, oral administration of a nano-lipid formulation (NLF) of our parent compound CFM-4 (CFM-4 NLF) resulted in significant improvement in its bioavailability over that noted for the orally administered, free CFM-4 compound [12]. Oral administration of CFM-4 NLF also inhibited growth of TNBC as well as non-small cell lung cancer cell-derived xenografts *in vivo* [12]. On the basis of these prior findings, a nano-lipid formulation of CFM-4.16 (CFM-4.16 NLF) was prepared and tested for its bioavailability and efficacy *in vivo* as detailed in methods. Oral administration of CFM-4.16 NLF resulted in a significant increase in the bioavailability when compared to CFM-4.16 Free drug (Figure 8A). The plasma Cmax concentration of CFM-4.16 free drug was found to be 1.19 ± 0.035 μg/ml. The Cmax concentration of CFM-4.16 NLF was 4.32±0.23 μg/ml, which was a 3.63 fold increase when compared with CFM-4.16 free drug. The AUC for CFM-4.16 Free drug was 21.07±4.20 μg.h/ml, whereas for CFM-4.16 NLF it was 86.21±17.20 μg.h/ml. The AUC for CFM-4.16 NLF was 4.09-fold more compared to CFM-4.16 Free drug. A 2.48-fold increased plasma half-life (t1/2) of CFM-4.16 NLF suggests for a sustained release behavior of CFM-4.16 NLF. Our studies therefore indicate that the improved pharmacokinetic parameters such as increased Cmax, t1/2 and AUC in the case of CFM-4.16 NLF led to its overall improved bioavailability over CFM-4.16 free drug by a 4.093-fold (Figure 8A).

The *in vivo* antitumor efficacy of CFM-4.16 NLF, ADR (noted as DOX), or their combination was investigated in TNBC MDA-MB-231 orthotropic xenograft tumor bearing nude mice as described in methods and our previously published studies [12]. As shown in Figure 8B, all the treatment groups showed significant tumor growth inhibition compared to the control (placebo) group. Although oral administration of CFM-4.16 NLF resulted in reduced breast tumor volume, a significantly higher reduction in the tumor volumes was noted in the CFM-4.16 NLF plus ADR (−DOX) group when compared with CFM-4.16 NLF or ADR (−DOX)-treated groups. These data indicate a statistically improved antitumor effect of combination compared to single drug groups (*p* < 0.05). Combination group showed 1.66 and 1.52 fold reduction in tumor volume compared to CFM-4.16 NLF and ADR (DOX) group, respectively (Figure 8B). Immuno-histological analysis of a representative TNBC tumor from the animals that were treated with placebo (control) or CFM-4.16 NLF plus ADR revealed increased staining for TUNEL and CARP-1 protein, and reduced staining for Oct4 (Figure 8C). The data in Figure 8 collectively demonstrate that NLF formulation of CFM-4.16 enhances its bioavailability, serum levels, and anti-tumor efficacy. In addition, CFM-4.16 inhibited xenografted TNBC tumors in part by diminishing levels of CSC-associated markers, and inducing apoptosis, and these findings would be consistent with our current *in vitro* observations as well as our previous studies where CFM-4 and CFM-4.6 compounds were found to stimulate apoptosis in a variety of cancer cell types including those of NSCLC and TNBC origins [8, 12–14].

## DISCUSSION

CFMs belong to an emerging class of novel scaffolds that function in part by inhibiting protein-protein interaction between CARP-1/CCAR1 and the E3 ubiquitin ligase Anaphase promoting complex subunit APC-2 [8]. The lead compound CFM-4 binds with CARP-1/CCAR1, causes elevated levels of CARP-1, stimulates apoptosis in a number of cancer cell types [8, 12–14], and oral administration of its nano-lipid formulation results in reduced growth of TNBC as well as non-small cell lung cancer (NSCLC) cell-derived xenografts *in vivo*. Given that frequent emergence of therapy-resistant TNBCs remains a significant and unresolved clinical problem, here we tested a hypothesis that potent analog(s) of CFM-4 scaffold are suitable inhibitors of TNBCs and their drug-resistant phenotypes *in vitro* and pre-clinical animal studies. As a first step in this direction, we found that out of twelve additional analogs of CFM-4 obtained by medicinal chemistry based structure activity relationship (SAR) studies (Table 1), two compounds, CFM-4.16 and CFM-4.17, possessed activities that were superior to the parent compound CFM-4 or other analogs. Interestingly, the compound CFM-4.16 in combination with ADR caused greater growth inhibition of only the TNBC cells when compared with either agent alone (Figure 1). On these bases, CFM-4.16 was chosen to test its potential as an inhibitor of TNBCs including the drug-resistant TNBCs.

Given that emergence of TNBCs that are resistant to chemotherapeutic drugs such as ADR and Cisplatin is a significant problem in clinic, we wished to investigate potential of CFM-4.16 and its class of scaffold as inhibitors of drug-resistant TNBCs. To test this hypothesis, we first developed a model by growing human and murine TNBC cells in continuous presence of chemotherapy (Cisplatin or ADR) over periods longer than 6 months, and obtained and characterized number of drug-resistant TNBC sublines. Indeed, the drug-resistant TNBC sublines had higher GI_50_ doses for the respective chemotherapeutic when compared with their parental, wild-type cells (see Table 2) indicating emergence of robust, drug-resistant TNBC phenotypes following step-wise dose escalation and chronic presence of the respective therapeutic. Importantly however, CFM-4.16 was effective in inhibiting viabilities of parental as well as drug-resistant TNBC cells in a dose-dependent manner (see Figure 2). Of note here is that CFM-4.16-dependent reduction in the viabilities of the drug-resistant cells was similar to that noted for the respective, CFM-4.16-treated parental cells, suggesting that molecular mechanisms of TNBC cell growth inhibition by CFM class of compounds are likely distinct from those utilized by chemotherapy such as ADR. Although ADR is known to require CARP-1 for apoptosis signaling in breast cancer including TNBC cells [4, 6], and ADR-resistant TNBC cells express elevated levels of CARP-1, CFM-4.16 exposure caused a further increase in CARP-1 levels in ADR-resistant cells (Figure 3). Moreover, similar to CFM-4, treatments of parental or ADR-resistant TNBC cells with CFM-4.16 stimulated activation of caspase-8, stress-activated kinases p38 and JNK1/2, and cleavage of PARP while inhibiting activation of oncogenic MET RTK, and causing reduced levels of cyclin B1 (Figures 3 and 4). Since depletion of CARP-1 in ADR-resistant TNBC cells also interfered with loss of their viabilities by CFM-4 or CFM-4.16 (Figure 3), collectively suggest for a requirement of CARP-1 for growth inhibition of ADR-resistant TNBC cells by CFM class of compounds. CFM-4 or CFM-4.16 also caused activation of stress and apoptosis signaling and CARP-1 increase in cisplatin-resistant TNBC and Herceptin-resistant, Her2-expressing SKBR-3 breast cancer cells (Supplementary Figure 1). Thus, CFM-4.16 although functions in part by activating CARP-1 and stress signaling to induce apoptosis in a manner that is analogous to CFM-4, the fact that CFM-4.16 but not CFM-4 enhances efficacy of ADR only in the TNBC cells would underscore its potential as a promising novel scaffold for development of agents to target drug-resistant TNBCs.

Both the MET and Src tyrosine kinases have been well known as drivers of carcinogenesis and development of resistance to therapies in many cancers including the TNBCs [22–25], and pre-clinical and clinical studies investigating benefits of MET or Src targeting have also been recently reported [26, 27]. Since MET activity was elevated in ADR or Cisplatin-resistant MDA-MB-468 cells, and Src activity was also elevated in ADR-resistant MDA-MB-468 and MDA-MB-231 cells (Figure 4, Supplementary Figure 3), would then be supportive of oncogenic roles of these kinases in drug-resistant TNBCs. Given that CFM-4.16 treatments caused diminished activities of MET in all the drug-resistant TNBC cells (Figure 4) while reducing Src in cisplatin-resistant TNBC cells only (Supplementary Figure 2), would be consistent with previous investigations supporting utility of targeting of these oncogenic kinases in TNBCs. Moreover, pre-clinical studies in a wide variety of solid tumors have shown that dasatinib is primarily cytostatic, and this is consistent with the clinical experience, where dasatinib activity is associated with stable disease but complete responses are rarely observed [28, 29]. Since pre-treatment of drug-resistant TNBC cells with CFM-4.16 resulted in significantly enhanced efficacies of MET or Src inhibitors (Figure 5), it would further argue for potential of CFM-4.16 scaffold to sensitize drug-resistant TNBCs to targeted therapies that are currently in the clinic (Src inhibitor Dasatinib) or under clinical development (MET inhibitor Tevatinib).

Our current studies further reveal that although drug-resistant TNBC cells have elevated expression of key regulators of stemness such as Klf4, Oct4, SOX2, cMyc and β-catenin (Figure 6), combination of CFM-4.16 and ADR cause diminished expression of Klf4, Oct4, and SOX2 in parental as well as ADR-resistant MDA-MB-231 cells. These findings would indicate that CFM-4.16 plus ADR are superior in targeting TNBC CSCs to prevent proliferation and differentiation of the small subset of stem-like populations. This possibility is further supported by our mammosphere studies where CFM-4.16 was effective in disrupting mammosphere structure of parental as well as drug-resistant TNBC cells. Collectively, our current *in vitro* studies demonstrate that although CFM-4.16 inhibits growth and survival of drug-resistant TNBC cells in part by stimulating apoptosis, its combination with ADR has unique ability to target pluripotent CSCs to suppress growth of parental and drug-resistant TNBC cells.

Given that a significant fraction of drug-like compounds that enter development programs often have poor aqueous solubility, and the fact that like CFM-4, CFM-4.16 also had poor aqueous solubility, prompted us to generate and test its nanolipid formulation for oral bioavailability and absorption. The nanolipid formulation incorporating a combination of two high melting solid lipid carriers (Compritol & Geleol) and the co-surfactant Vitamin E TPGS (with Tween-80) to promote sustained release, and stability and permeability of CFM-4.16, respectively. To prevent lipolysis of formulation due to lipid digestibility in GI tract and minimize drug escape from lipid carriers, liquid lipid “Miglyol” was added in the formulation. A natural, biocompatible cationic polysaccharide chitosan was also used to improve drug availability in tumor and interaction of positively charged formulation with negatively charged tumor surface. Indeed, CFM-4.16 exhibited good lipid solubility (Figure 8A), and CFM-4.16 NLF exhibited superior absorption as indicated by pharmacokinetic parameters Cmax and AUC values when compared to free compound.

Finally, our preclinical studies with TNBC cell-derived xenografts demonstrate therapeutic potential of CFM-4.16. Oral administration of CFM-4.16 NLF suppressed growth of xenografted TNBC tumors in nude mice with an efficacy that was comparable to that noted for intravenously administered ADR (DOX). Interestingly, a combination of CFM-4.16 NLF and ADR elicited a significantly superior suppression of xenograft growth when compared to that noted with each agent alone. The facts that elevated CARP-1 and apoptosis, and diminished Oct4, were noted in xenografts from animals treated CFM-4.16 NLF plus ADR but not in control, untreated animals (Figure 8C), corroborate our current *in vitro* observations and highlight a unique property of CFM-4.16 to enhance ADR efficacy in part by targeting CSCs for suppressing TNBCs.

In conclusion, our studies report identification of a novel CFM analog that inhibits viabilities of parental and drug-resistant TNBC cells *in vitro*. In addition to inhibiting expression and/or activation of various growth and survival-promoting genes, CFM-4.16 inhibited expression of proteins associated with stem-like cells. CFM-4.16 in combination with TKIs (Dasatinib or Tivatinib) caused greater loss of viabilities of parental as well as ADR-resistant TNBC cells. Pre-treatments with CFM-4·16 increased ADR-dependent inhibition of cell viability of only the TNBC cells *in vitro*, while a combination of ADR and CFM-4.16 elicited superior inhibition of growth of TNBC cell-derived xenografts *in vivo* when compared with either agent alone.

## MATERIALS AND METHODS

### Cells and reagents

Routine culture and maintenance of human TNBC cell lines MDA-MB-468, MDA-MB-231, HCC1937, and Hs578T, the SKBR-3 human breast cancer (HBC) cells that lack estrogen receptor, have mutant p53, and overexpress Her-2, cervical cancer HeLa, and human malignant pleural mesothelioma (MPM) H2461 and H2373 was carried out essentially as described before [8, 12]. Human TNBC CRL2335, BT-20, and HCC-1806 cells were purchased from ATCC, and were kindly provided by Drs. Julie Boerner, and Kaladhar Reddy Departments of Oncology and Pathology, respectively, Wayne State University, Detroit, MI. The CRL2335, BT-20, and HCC-1806, were routinely cultured essentially as described before [12, 30]. Herceptin-resistant SKBR-3 HBC cells were kindly provided by Dr. Rita Nahta, Emory University Cancer Center, Atlanta, GA, and cultured essentially as described [31]. 4T1, a highly metastatic murine breast cancer cell line derived from a spontaneously arising BALB/c mammary tumor were obtained from the Karmanos Cancer Institute (KCI) and maintained essentially as described before [32, 33]. The human umbilical vein endothelial cells (HUVECs) and the *in vitro* angiogenesis assay kit was purchased from Lonza Walkersville Inc., Walkersville, MD and Chemicon International Inc., Temicula, CA, respectively. The HUVECs were maintained in a specified media (EGM Bullet kit; Lonza Walkersville Inc.) per the manufacturer suggested guidelines. All the cell culture media were also supplemented with 10% FBS, 100 units/ml of penicillin, and 100 μg/ml of streptomycin, and the cells were maintained at 37°C and 5% CO_2_ [8, 12, 13].

DMEM, RPMI-1640 medium, penicillin and streptomycin were purchased from Invitrogen Co. (Carlsbad, CA), and dimethyl sulfoxide (DMSO) was purchased from Fischer Scientific (Fair Lawn, NJ). FBS was purchased from Denville Scientific Inc. (Metuchen, NJ), and 3–4, 5-dimethyltiazol-2-yl-2.5-diphenyl-tetrazolium bromide (MTT), research grade Cisplatin, and Anti-β-actin mouse monoclonal antibody were purchased from Sigma-Aldrich (St. Louis, MO). Cisplatin was dissolved in phosphate buffered saline. Enhanced Chemiluminescence Reagent was purchased from Amersham Biosciences (Piscataway, NJ) and the Protein Assay Kit was purchased from Bio-Rad Laboratories (Hercules, CA). CFM-4 was obtained from ChemDiv, San Diego, and was dissolved in DMSO at a stock concentration of 50 mM and stored at −20°C. Clinical grade Adriamycin (ADR), Cisplatin, and Herceptin were obtained from Karmanos Cancer Institute Pharmacy, Detroit, MI while the research grade ADR along with dual Src and Bcr-Abl inhibitor Dasatinib, and c-Met inhibitor Tevatinib were purchased from SelleckChem, Boston, MA and dissolved in manufacturer suggested solvent (water or DMSO) to obtain appropriate stocks that were stored at −20°C until needed.

Generation and characterization of the anti-CARP-1/CCAR1 rabbit polyclonal antibodies have been described elsewhere [4]. The mouse monoclonal antibodies for α-tubulin and βCatenin were obtained from Calbiochem and Millipore (Billerica, MA), respectively. Anti-cyclin B1, anti phospho-JNK (Threonine183/Tyrosine185) G9, caspase-8, and cleaved PARP mouse monoclonal antibodies, phospho-STAT3 (Y705), phospho-MKK4 (S257), total STAT3, Klf4, Sox2, anti-MET, c-myc, anti-JNK (56G8) rabbit monoclonal antibodies, and phospho-MET (Y1234/1235), Oct4, AKT, PARP, mToR, p70S6K, MKK4, phospho and total p38α/β SAPK rabbit polyclonal antibodies were obtained from Cell Signaling Technology (Beverly, MA). The On-Target plus SiRNAs for knockdown of CARP-1/CCAR1 were purchased from Dharmacon (ThermoFisher).

### Chemical synthesis of CFM-4 analogs

Data from an initial SAR survey of 35 commercially available analogs of CFM-4 was used to guide the design of additional analogs. Optimal R1, X and R2 substitutions on the CFM-4 template established to date are presented in Table 1. Several R3 substituents were also generated on the aromatic ring (Cl, Br, alkyl and NO2), and all were well tolerated. Synthesis of structural analogs of CFM-4 from diverse isatins and thiosemicarbazides was carried out essentially as described before [34] followed by their screening for biological activity in cells *in vitro* by MTT assays below.

### Generation of drug-resistant TNBC cells

Human TNBC MDA-MB-468 and MDA-MB-231, and mouse TNBC 4T1 cells were cultured in the chronic presence (> 10 months) of Doxorubicin. The parental, wild-type cells were initially treated with 200 nM Doxorubicin for 2–3 weeks, followed by escalation to 400 nM, 1 μM, and 2 μM doses over a period of 3–4 weeks for each dose till resistance developed and the cells became well adapted to growth in 1 μM dose of Doxorubicin for their routine culture. In the case of Cisplatin however, the human TNBC MDA-MB-468 and MDA-MB-231 were initially cultured in continuous presence of 1 μM Cisplatin for 3–4 weeks, and the dose was escalated to 1.5 μM and 3 μM over periods of 3–4 weeks for each dose till resistance developed and cells became adapted to routine culture in 3 μM dose. Subsequent, routine maintenance of the resistant cells in the presence of the respective drug was continued and multiple, resistant sublines for each of the TNBC cells were isolated and characterized for their growth inhibitory (GI)_50_ dose of respective therapeutic by the MTT-based viability assays as below.

### MTT and western blot assays

*In vitro* inhibition of cell growth was assessed by MTT (3-[4, 5-dimethyltiazol-2-yl]-2.5-diphenyl tetrazolium bromide) reagent. Cells (5 × 10^3^) were seeded in a 96-well culture plate and subsequently treated with respective agents at different concentrations as mentioned. Control cells were treated with 0.1% DMSO in culture medium. After treatment, the cells were incubated with 1 mg/ml of MTT reagent at 37°C for 2–4 hours and then MTT was removed and 50 μL of DMSO was added, followed by colorimetric analysis using a multi-label plate reader at 560 nm (Victor3; PerkinElmer, Wellesley, MA, USA).

For protein expression analysis, Western blot (WB) experiments were performed according to the standard procedures. The cells were either untreated or treated with various agents either alone or in combinations as indicated. Following treatment durations, the cells were lysed in cell lysis (10X) buffer (#9803; cell signaling) containing 0.1% of protease and phosphatase inhibitor cocktail (Sigma) for 20 min at 4°C. The lysates were centrifuged at 14,000 rpm at 4°C for 15–20 min to remove debris. Protein concentrations of whole cell lysates were determined using the Protein Assay Kit. Supernatant proteins, 50–100 μg from each sample, were separated by SDS-polyacrylamide gel electrophoresis (SDS-PAGE) and transferred to polyvinylidene difluoride (PVDF) membrane (Bio-rad, Hercules, CA) by standard procedures. The membranes were hybridized with primary antibodies followed by incubation with appropriate secondary antibodies using manufacturer suggested dilutions. The antibody-bound proteins were visualized by treatment with the chemiluminescence detection reagent according to manufacturer's instructions, followed by exposure to X-ray film (Denville Scientific Inc.). The same membranes were re-probed with the anti-β-actin or anti-α-tubulin antibody, which was used as an internal control for protein loading.

### Cell migration and clonogenic assays

The TNBC cells migration in the absence or presence of CFMs was measured by the “scratch/ wound healing” assay. Cells were seeded in a 6-well plate (~10,000 cells/well), and when attached, a scratch was created in the cell monolayer using sterile pipette tip. The cells were then allowed to continue growing in the absence (Control) or presence of noted dose of each of the agents for indicated time periods. The cells were photographed at the beginning and at regular intervals during the treatment period, and the images from control cells were compared with the treated cells to determine the migration of the cells essentially as described before [35]. The photomicrographs of the cells were recorded under different magnifications utilizing Zeiss microscope with attached 35 mm camera.

### Clonogenic assay

A soft-agar sandwitch assay was performed. Cells were sandwiched between 0.6% and 0.3% agarose in DMEM medium containing 5% FBS in a six-well chamber (500 cells/chamber), and treated with buffer (Control), or respective agent for noted time and dose at 37°C humidified CO_2_ incubator. The colonies from multiple random fields were counted, compared to control and photographed essentially as described before [13, 14, and 36].

### Formulation of CFM-4.16 nano lipid carriers (CFM-4.16 NLF) and pharmacokinetic studies

Preparation and characterization of CFM-4.16 NLF was carried out essentially as described [12]. Briefly, appropriate amounts of CFM-4.16 were blended with Compritol 888ATO, Miglyol 812N, and Geleol, and the mixture was melted at 70°C to form a uniform and clear oil phase. Next, an aqueous phase consisting of dispersing surfactant Tween 80 and Vitamin E TPGS in double distilled water was added drop wise to the oil phase at 70°C and phases agitated at 5000 rpm for 5 min using tissuemiser. The coarse emulsion was then homogenized for 15 min under high pressure. The NLF preparation was then processed with NanoDebee for about 5 cycles followed by probe sonication for 5 min to reduce its size [12, 37].

Further surface modification of NLF preparation was carried out by mixing with appropriate amounts of the chitosan polymer (CP) essentially as described before [12, 38]. Briefly, CP was dissolved in water to obtain a series of concentrations (0.25%, 0.5%, 1%, 2%, w/v), and then mixed with CFM-4 NLF dispersions. In each case, an aliquot of NLF was mingled with an equal volume of CP by adding it drop wise under continuous agitation at room temperature (20°C) over a 30 min incubation period. The surface modified CFM-4.16 NLF formulation was subjected to drug encapsulation efficiency, measurement of particle size and zeta potential, and *in vitro* drug release studies following methods describe by us before [12].

Pharmacokinetic Studies were performed in rodents (Sprague Dawley Rats) to determine the bio-availability kinetics of the CFM-4.16 NLF formulation and CFM-4.16 free drug (FD) following previously detailed protocols [12]. Briefly, rats were fasted overnight prior to the start of experiments and randomly divided into three experimental groups receiving CFM-4.16 FD and CFM-4.16 NLF at 40 mg/kg orally and CFM-4.16 solution (CFM-4.16 sol) at 5 mg/kg by intravenous route. After the drug administration, blood samples (250 μl) were withdrawn from tail veins at 0, 0.25, 0.5, 1, 2, 4, 6, 8, 12 and 24 h, and collected directly into heparinized microvet blood collection tubes and plasma was obtained by centrifugation at 10,000 rpm for 10 min. The plasma samples were stored at −80°C until needed for analysis. CFM-4.16 was extracted from the plasma by protein precipitation method and extracted samples were dissolved in mobile phase and samples were analyzed by HPLC. Oral bioavailability of CFM-4.16 FD and CFM-4.16 NLF along with their pharmacokinetic parameters such as area under curve (AUC), Cmax, t1/2, and tmax were estimated using non-compartmental techniques with WinNonlin^®^ 5.0 software (Pharsight Corporation, Mountain View, CA, USA).

### Establishment of TNBC cell-derived xenografts in immunocompromised mice

The experiments involving generation of TNBC cell-derived sub-cutaneous xenografts were performed according to our previously published methods and protocols approved by the Institutional Laboratory Animal care & Use Committees at the Wayne State and Florida A&M Universities [12, 37, 39]. Female, 5-weeks old NCR SCID mice with Lc Background or Balb/c nude mice were purchased from Charles River Laboratories (Horsham, PA). The orthotopic TNBC xenograft studies were carried out in female BALB/c Nude Mice. Following suitable acclimation of animals, 1 × 10^6^ MDA-MB-231 TNBC cells were re-suspended in 200 μl of serum-free Hank's balanced salt solution, and implanted in the mammary fat pads using a 27-gauge needle. Tumors were allowed to grow unperturbed for 10–14 days. When tumors became palpable (200 mm^3^), the mice were randomly assigned to treatment or control groups of six animals each. Mice were treated with Control, PBS only, CFM-4.16 NLF (40 mg/kg), Doxorubicin (5 mg/kg), or CFM-4.16 NLF + Doxorubicin every alternate day for 2 weeks. CFM-4.16 NLF was administered by oral gavage while Doxorubicin was given by intravenous route by tail vein. One week after the last dose of drugs, animals were sacrificed and tumor tissues were collected immediately after tumor volume measurement. Tumor volumes were calculated by the modified ellipsoidal formula. Tumor volume = 1/2(length × width^2^). Representative tumor samples were stored at −80°C for subsequent analysis.

For subcutaneous (sc) tumor xenograft studies, first a maximal tolerated dose for CFM-4.16 was determined in the NCR-SCID mice. For this purpose, CFM-4.16 prepared in 10% DMSO/cremophor + distilled, sterile water, and pH was adjusted to 4.5. This preparation was injected daily iv at 24 mg/kg on days 1 and 2, 30 mg/kg on days 3–9 and at 32 mg/kg on days 10–16 (some mice were switched to SC route due to swollen tail veins days 12–16) for a total dose of 482 mg/kg. A mild ataxia with some tail and leg twitching resolving within 1–2 minutes was seen. This dose/schedule produced a mild weight loss of 1.6% body weight by day 7(recovery by day 18). No other histological abnormalities were noted. Subcutaneous tumor xenografts from MDA-MB-231 TNBC cells were generated in female NCR-SCID mice as described before [12]. When the tumors developed, mice were sacrificed, tumors were dissected, cut into small fragments and either stored in liquid N2 or dissociated to isolate tumor-derived parental and drug-resistant human TNBC cells that were subsequently cultured for 3-dimensional mammosphere assays as below. In addition, tumor fragments were transplanted sc into similarly conditioned animals (*n* = 10 for each group) by using a 12-guage trocar. Mice were checked 3-times a week for tumor development. Once palpable tumors developed, the groups of animals were subjected to efficacy trial with CFM-4.16. The animals bearing xenografts were either untreated (Control group; 10 mice) or treated with 470 mg/kg dose of CFM-4.16 (Qd 1–19; iv administration). The tumor measurements were carried out at multiple time points during the course of treatment and observation periods. Mice were observed for changes in weight and side effects followed by measurement of tumors three times per week. The end points for assessing antitumor activity consisted of tumor weight, tumor growth inhibition (%T/C), and tumor cell kill Log10. Tumor weight (mg) = (A X B2)/2 where A and B are the tumor length and width (in mm), respectively. Tumor growth inhibition (T/C) was the median tumor weight in the treated group (T) when the median tumor weight in the control group reached 750 mg. Results was expressed as percentage. According to NCI-accepted criteria, a treatment is considered effective if T/C is < 42%. Tumor growth delay (T-C) is the difference between the median time (in days) required for the treatment group tumors (T) to reach 750 mg and the median time (days) for the control group tumors to reach the same weight.

### Three-dimensional mammosphere assays

The TNBC cells were obtained from tumors derived from parental and drug-resistant cells or from a two-dimensional culture plate with ~70–80% confluence. The cells were washed twice in 1 × PBS and trypsinized following established protocols. The cells were pelleted at 200 × g at room temperature, and re-suspended in 5 ml of mammosphere media (DMEM/F12 supplemented with 2 mM L-glutamine, 100 U/ml penicillin, 100 U/ml streptomycin, 1 × B27 supplement, 20 ng/ml recombinant human epidermal growth factor (EGF; Sigma), 10 ng/ml recombinant human basic fibroblast growth factor (bFGF; R&D Systems). The cell suspension of ~5000 viable cells per ml was then seeded in a ultra-low adherent 60 mm plate and incubated at 37°C and 5% CO_2_ for two weeks without disturbing the plates. After the mammospheres formed, fresh media with or without 10 μM CFM-4.16 was added and the cells incubated for additional 24 h at 37°C and 5% CO_2_. The mammospheres in the untreated and treated plates were photographed, and the cells were then dissociated to determine their viabilities by the MTT assay as described [40].

### Statistical analysis

In some instances, statistical analysis was performed using unpaired Student's *t-test*. A *p-value* less than 0.05 between treatment groups was considered significantly different.

## SUPPLEMENTARY MATERIALS FIGURES


