# Supplementary material for: CARP-1 functional mimetics are novel inhibitors of drug-resistant triple negative breast cancers
Source: Oncotarget. 2016 Sep 28;7(45):73370–88. doi: 10.18632/oncotarget.12333 (PMC5341985; doi:10.18632/oncotarget.12333)
Supplement: Supplementary file 1 [file oncotarget-07-73370-s001.pdf]

CARP-1 functional mimetics are novel inhibitors of drug-resistant triple negative breast cancers

Supplementary Materials

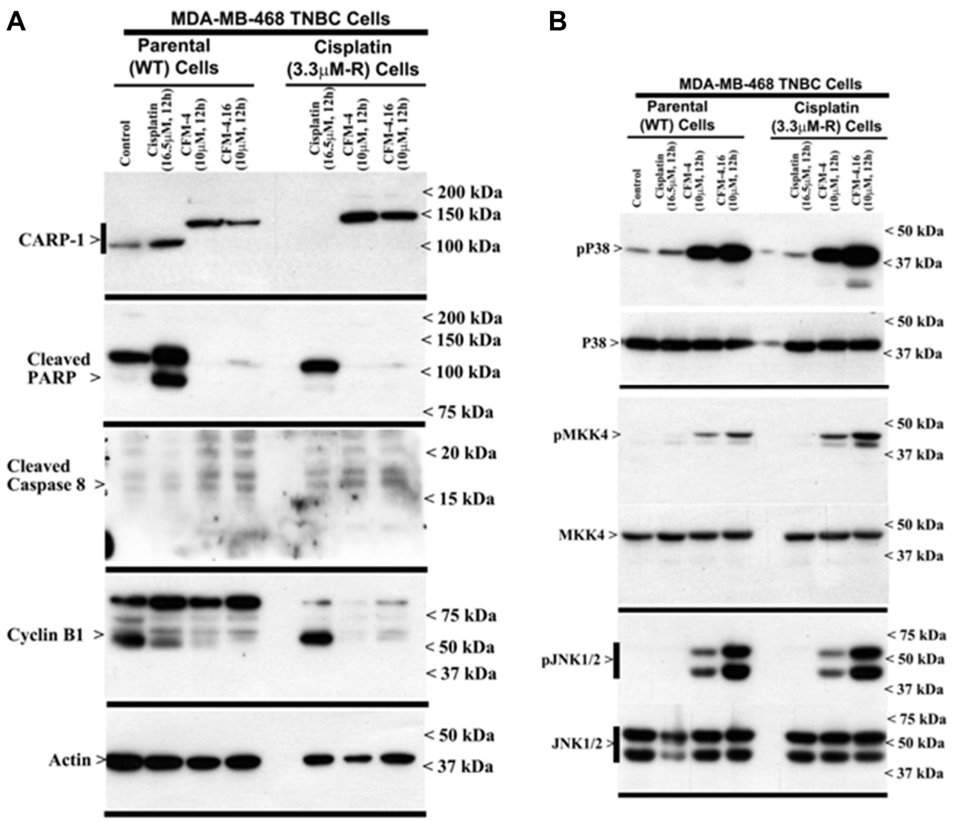

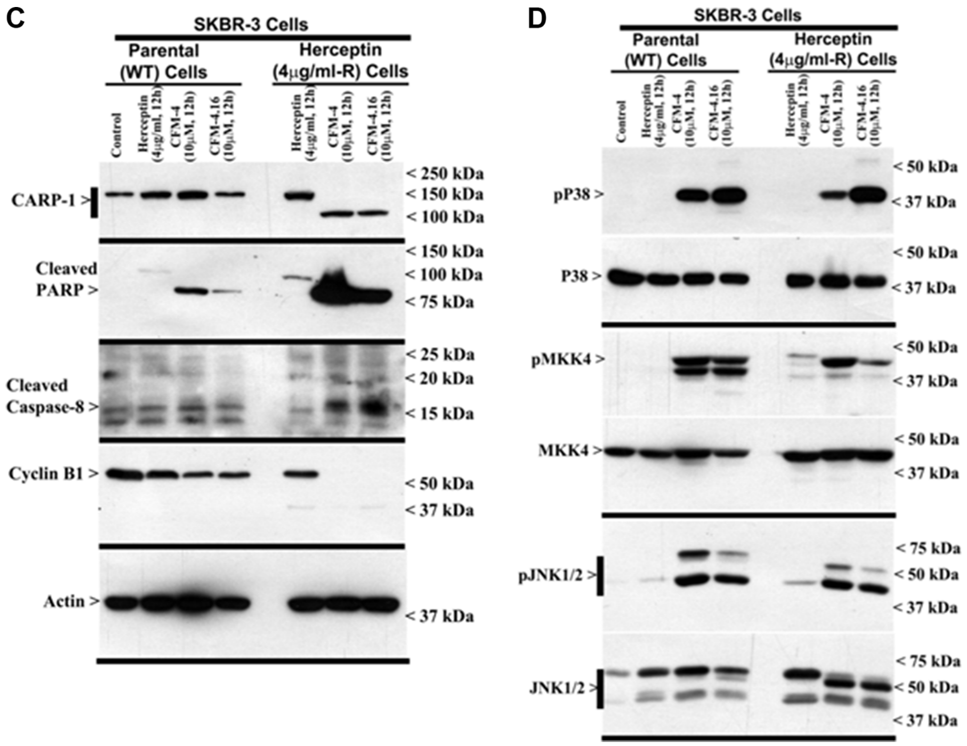

Supplementary Figure S1: CFM-4.16 stimulates apoptosis in parental and Cisplatin-resistant TNBC cells (A, B) as well as in Herceptin-resistant breast cancer cells (C, D) in part by upregulating pro-apoptotic CARP-1. Indicated breast cancer cells were either untreated (Control), treated with Cisplatin, Herceptin, CFM-4, or CFM-4.16 for noted dose and time. Cell lysates were analyzed by Western blotting as in Methods for levels of CARP-1, cyclin B1, cleaved PARP and caspase-8 (A, C) and activation (phosphorylation) of pro-apoptotic p38, MKK4, and JNK1/2 SAPKs (B, D).

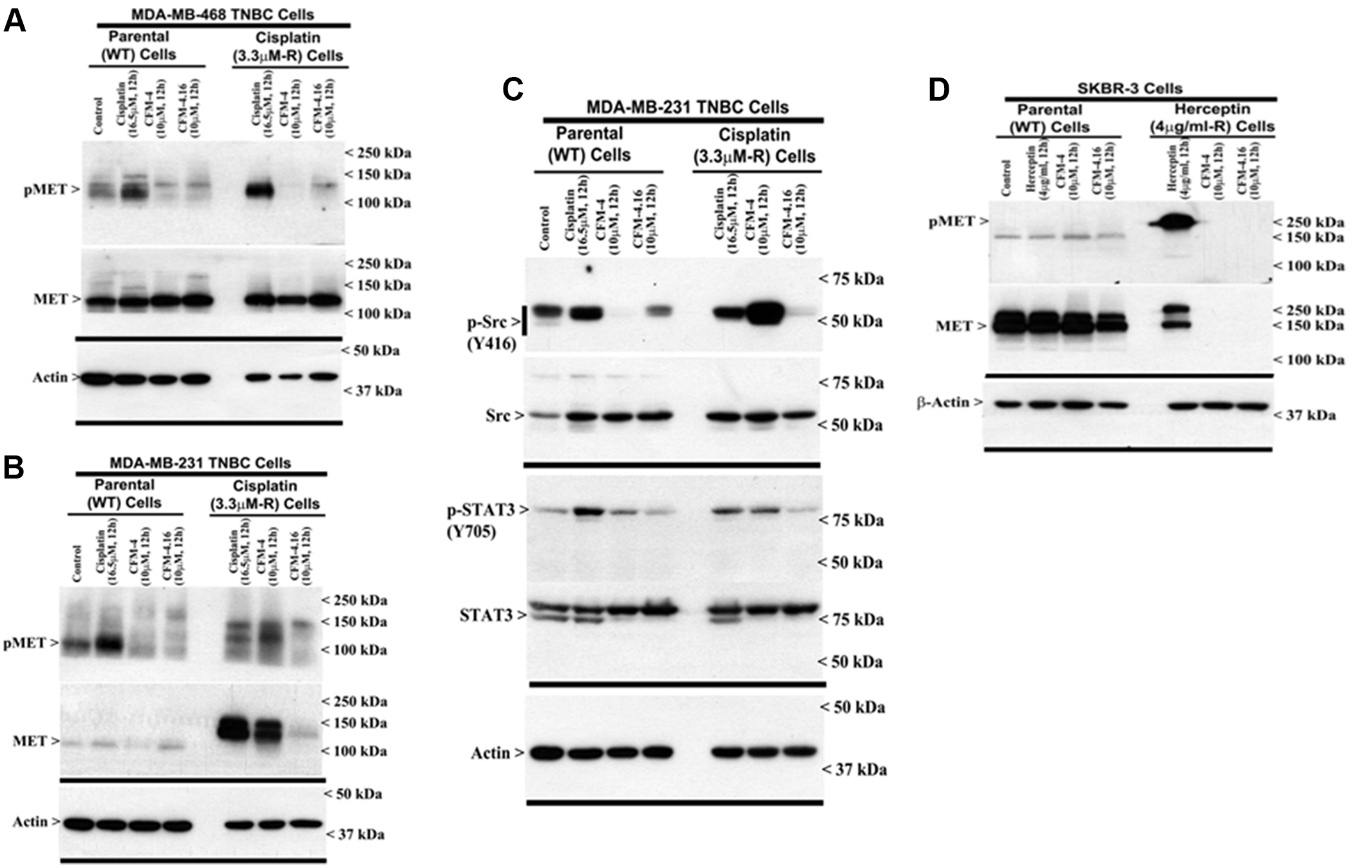

Supplementary Figure S2: CFM-4.16 inhibits oncogenic tyrosine kinases in Cisplatin-resistant TNBC cells (A–C) as well as in Herceptin-resistant breast cancer cells (D). Indicated breast cancer cells were either untreated (Control), treated with Cisplatin, Herceptin, CFM-4, or CFM-4.16 for noted dose and time. Cell lysates were analyzed for expression and activation (phosphorylation) of Src, MET, and STAT3 kinases, and levels of actin proteins by Western blotting as described in Methods. Identity of respective protein and molecular weight markers is denoted by arrowheads on the left and right side, respectively, of each WB.

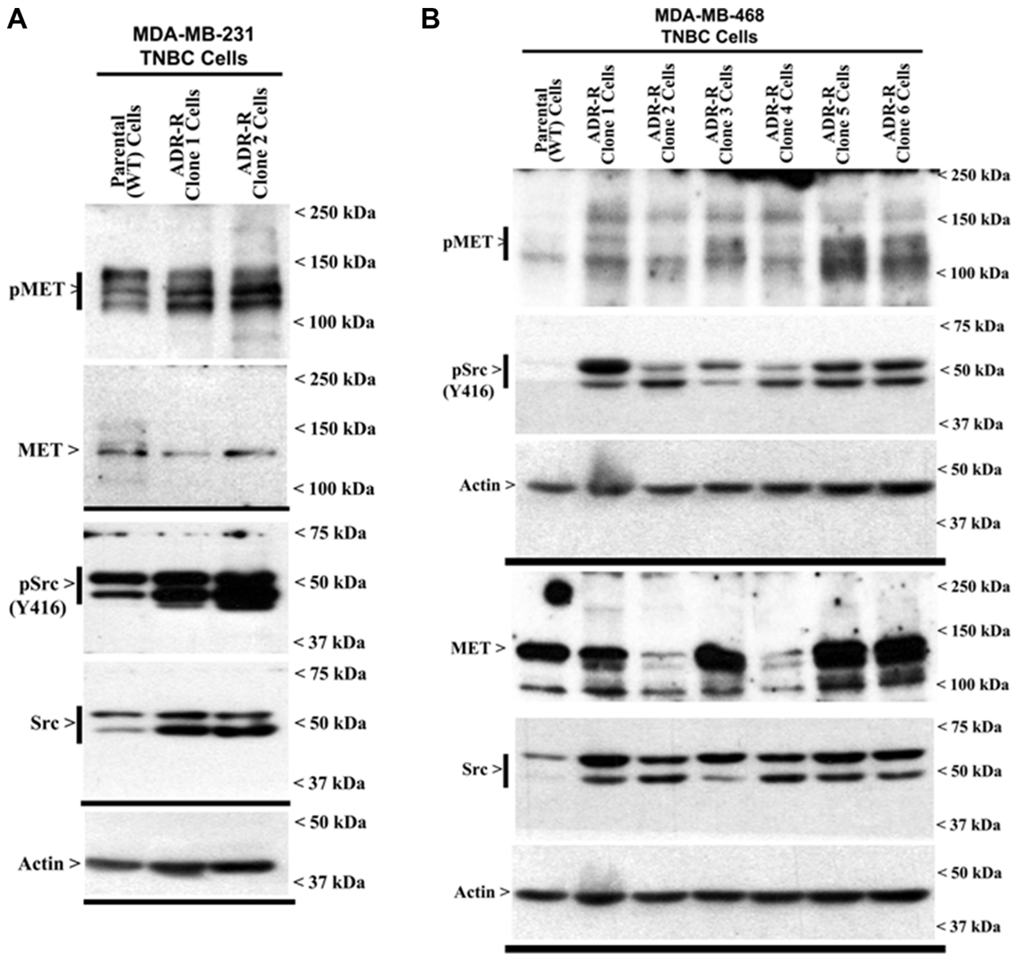

Supplementary Figure S3: Activation and expression of Src and MET tyrosine kinases in parental and ADR-resistant TNBC cells. Cell lysates from indicated TNBC cells were analyzed for expression and activation (phosphorylation) of Src, MET, and levels of actin proteins by Western blotting as described in Methods. Identity of respective protein and molecular weight markers is denoted by arrowheads on the left and right side, respectively, of each WB. Please note that in the case of MDA-MB-468 cells, the lysates were analyzed on two separate gels. Following transfer of proteins, one membrane was incubated with antibodies for p-Src, p-MET, and Actin proteins while the second membrane was incubated with antibodies for total Src, MET, and actin proteins.

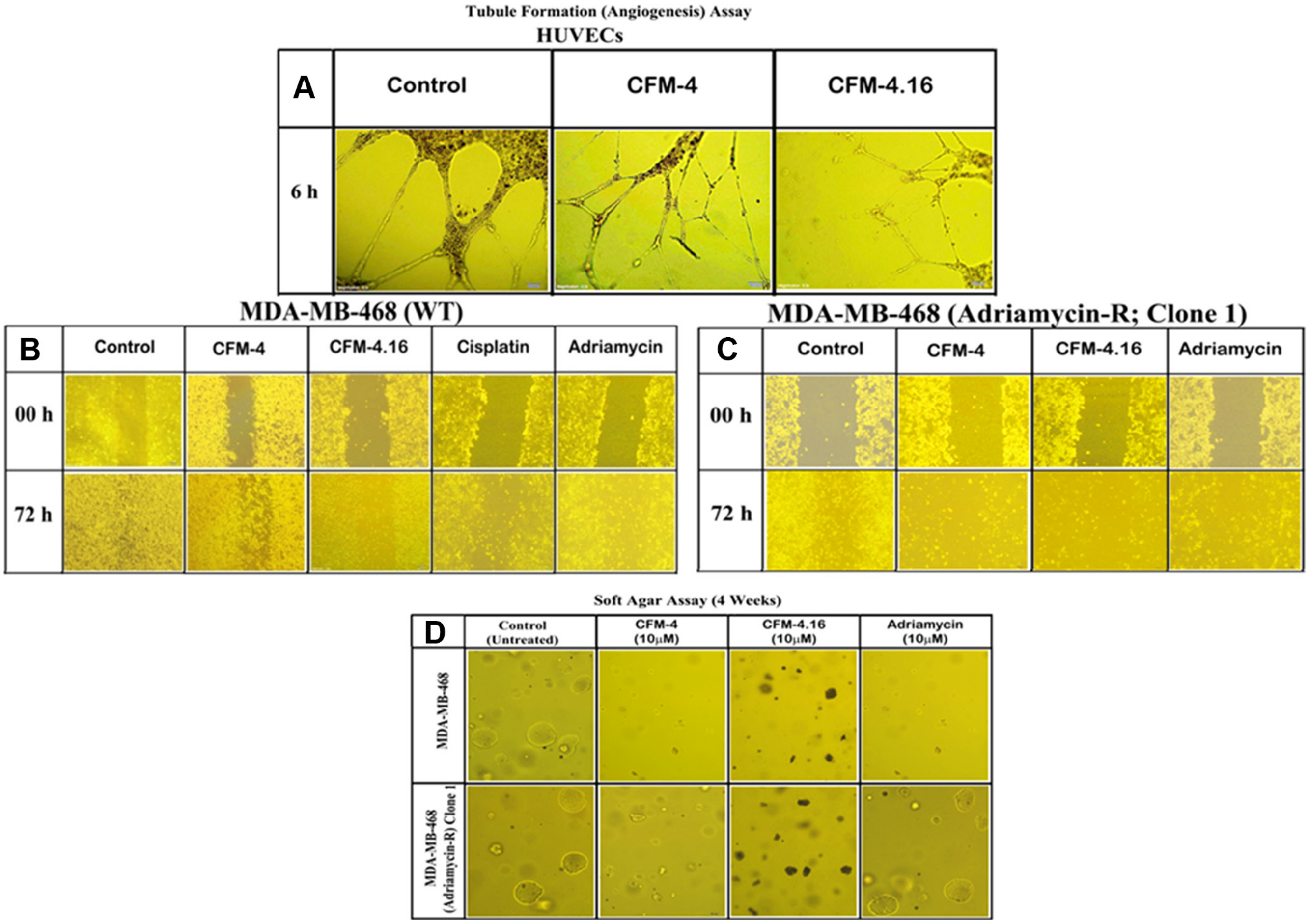

Supplementary Figure S4: CFM-4.16 inhibits angiogenesis, parental and ADR-resistant TNBC cell motility, and growth in soft agar. (**A**) HUVECs were seeded onto a surface containing polymerized extracellular matrix (ECM), and allowed to develop network structures in buffer (Control) or CFM-treated cells. The growth of tubules was monitored over a period of 12 hours. Representative photomicrographs of control and treated cells are presented. (**B, C**), Indicated TNBC cells were either untreated (Control), treated with 3 μM of respective CFMs, 3.3 μM of cisplatin, or 1.0 μM of ADR for 72 h, and subjected to scratch assays as described in Methods. (**D**), Indicated TNBC cells were seeded in soft-agar and either untreated (Control), treated with 10 μM of CFM-4, CFM-4.16, or ADR for noted time. The cells growth in the scratch assay in panels B, C or the number of colonies of cells in panel D were recorded by photography as detailed in Methods. Representative photomicrographs of untreated and treated TNBC cells are shown.

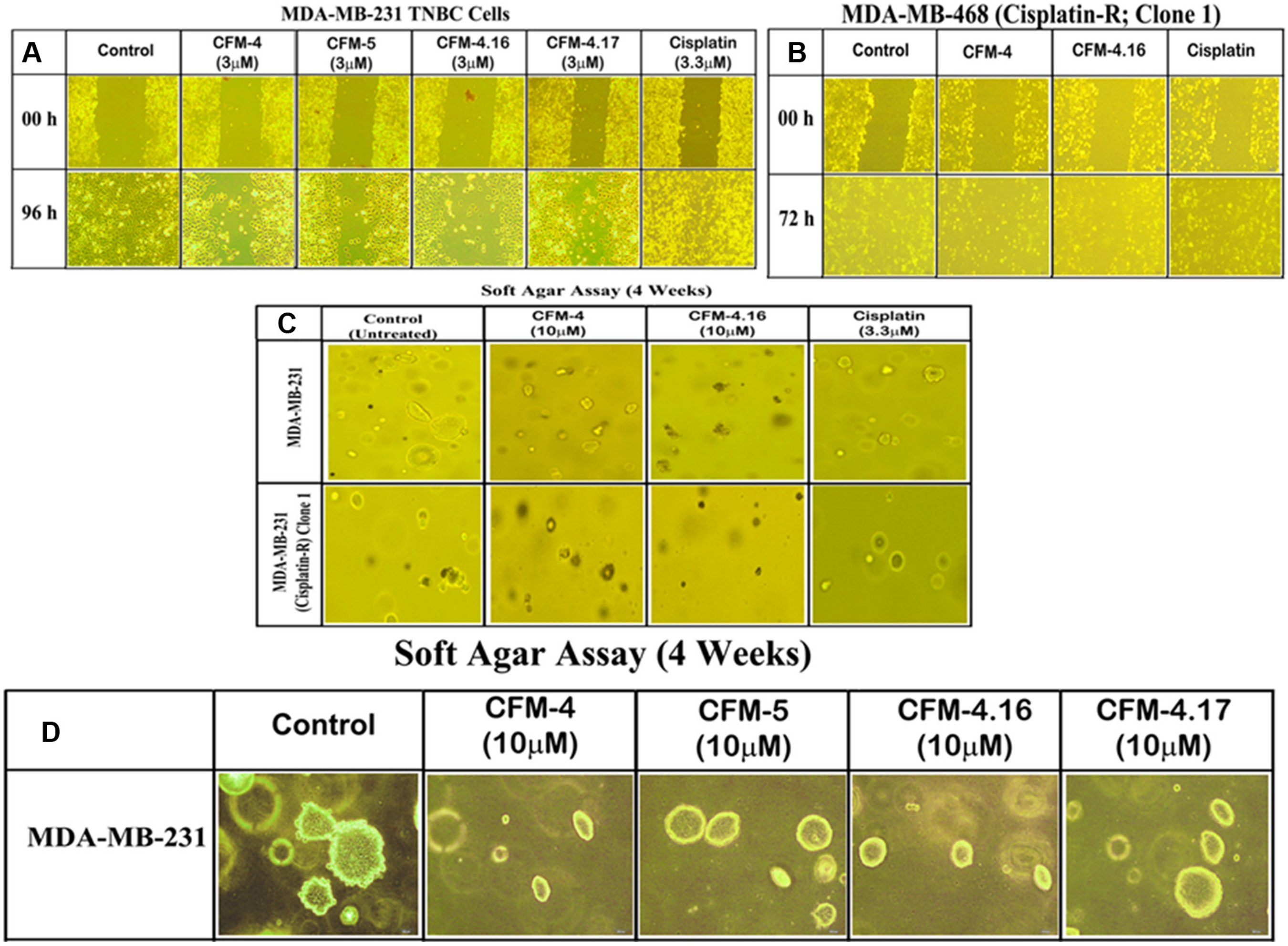

Supplementary Figure S5: CFM-4.16 inhibits parental and Cisplatin-resistant TNBC cell motility, and growth in soft agar. (**A, B**) Indicated TNBC cells were either untreated (Control), treated with 3 μM of respective CFMs or 3.3 μM of cisplatin for noted times, and were subjected to scratch assays as described in Methods. (**C, D**), Indicated TNBC cells were seeded in soft-agar and either untreated (Control), treated with 10 μM of each of CFMs, or 3.3 μM of Cisplatin for noted time. The cells growth in the scratch assay in panels A, B or the number of colonies of cells in panels C, D were recorded by photography as detailed in Methods. Representative photomicrographs of untreated and treated TNBC cells are shown.

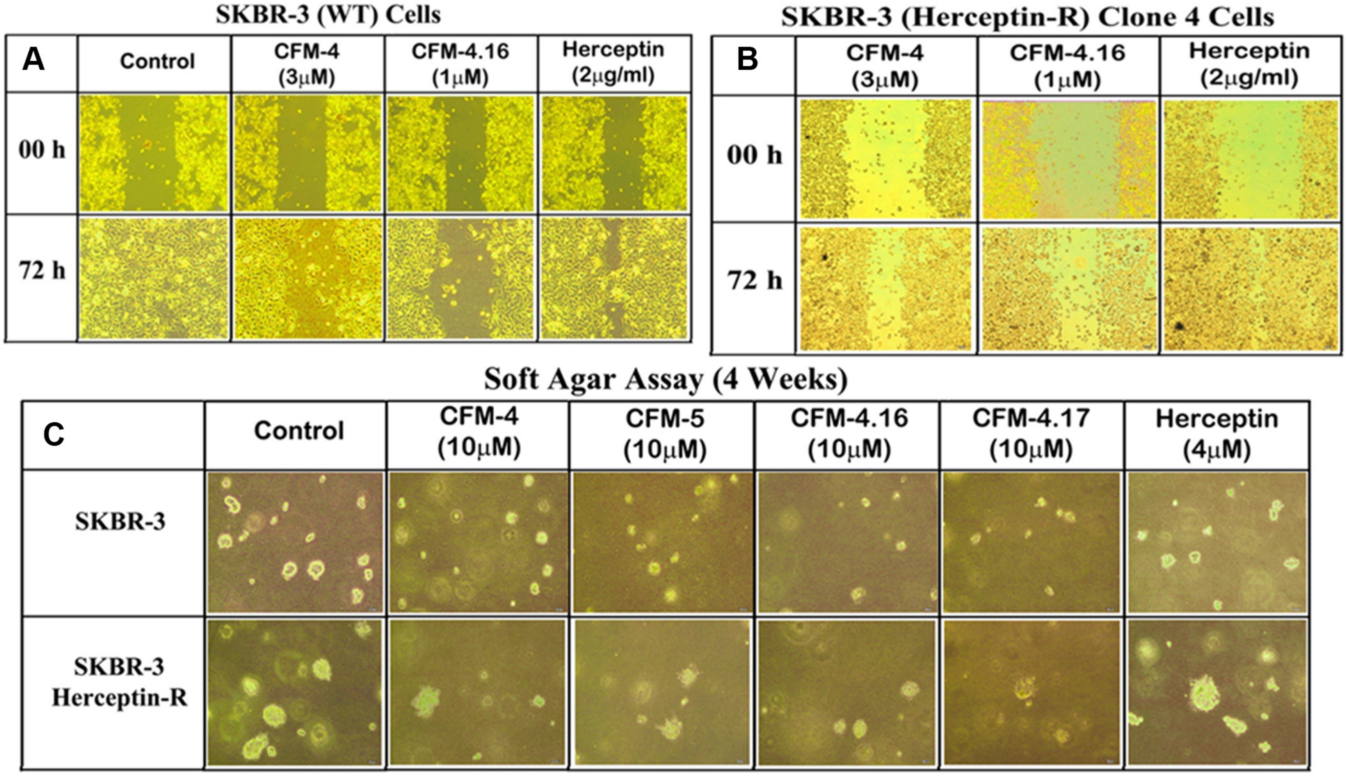

Supplementary Figure S6: CFM-4.16 inhibits parental and Herceptin-resistant breast cancer cell motility, and growth in soft agar. (**A, B**) Indicated breast cancer cells were either untreated (Control), treated with 3 μM of CFM-4, 1.0 μM of CFM-4.16, or 2 μg/ml Herceptin for noted times, and were subjected to scratch assays as described in Methods. (**C**), Indicated breast cancer cells were seeded in soft-agar and either untreated (Control), treated with 10 μM of each of CFMs, or 4 μM of Herceptin for noted time. The cells growth in the scratch assay in panels A, B or the number of colonies of cells in panel C were recorded by photography as detailed in Methods. Representative photomicrographs of untreated and treated breast cancer cells are shown.
